# A Bat-Derived Putative Cross-Family Recombinant Coronavirus with a Reovirus Gene

**DOI:** 10.1371/journal.ppat.1005883

**Published:** 2016-09-27

**Authors:** Canping Huang, William J. Liu, Wen Xu, Tao Jin, Yingze Zhao, Jingdong Song, Yi Shi, Wei Ji, Hao Jia, Yongming Zhou, Honghua Wen, Honglan Zhao, Huaxing Liu, Hong Li, Qihui Wang, Ying Wu, Liang Wang, Di Liu, Guang Liu, Hongjie Yu, Edward C. Holmes, Lin Lu, George F. Gao

**Affiliations:** 1 National Institute for Viral Disease Control and Prevention, Chinese Center for Disease Control and Prevention (China CDC), Beijing, China; 2 College of Laboratory Medicine and Life Sciences, Wenzhou Medical University, Wenzhou, China; 3 Yunnan Provincial Center for Disease Control and Prevention, Kunming Yunnan, China; 4 China National Genebank-Shenzhen, BGI-Shenzhen, Shenzhen, China; 5 CAS Key Laboratory of Pathogenic Microbiology and Immunology, Institute of Microbiology, Chinese Academy of Sciences, Beijing, China; 6 Center for Disease Control and Prevention of Mengla County, Mengla Yunnan, China; 7 CAS Key Laboratory of Microbial and Metabolic Engineering, Institute of Microbiology, Chinese Academy of Sciences, Beijing, China; 8 Network Information Center, Institute of Microbiology, Chinese Academy of Sciences, Beijing, China; 9 Division of Infectious Disease, Key Laboratory of Surveillance and Early-warning on Infectious Disease, Chinese Centre for Disease Control and Prevention, Beijing, China; 10 Marie Bashir Institute of Infectious Diseases and Biosecurity, Charles Perkins Centre, School of Biological Sciences and Sydney Medical School, The University of Sydney, Sydney, New South Wales, Australia; 11 Laboratory of Protein Engineering and Vaccines, Tianjin Institute of Industrial Biotechnology, Chinese Academy of Sciences, Tianjin, China; 12 Research Network of Immunity and Health (RNIH), Beijing Institutes of Life Science, Chinese Academy of Sciences, Beijing, China; 13 Office of Director-General, Chinese Center for Disease Control and Prevention (China CDC), Beijing, China; Loyola, UNITED STATES

## Abstract

The emergence of severe acute respiratory syndrome coronavirus (SARS-CoV) in 2002 and Middle East respiratory syndrome coronavirus (MERS-CoV) in 2012 has generated enormous interest in the biodiversity, genomics and cross-species transmission potential of coronaviruses, especially those from bats, the second most speciose order of mammals. Herein, we identified a novel coronavirus, provisionally designated Rousettus bat coronavirus GCCDC1 (Ro-BatCoV GCCDC1), in the rectal swab samples of *Rousettus leschenaulti* bats by using pan-coronavirus RT-PCR and next-generation sequencing. Although the virus is similar to Rousettus bat coronavirus HKU9 (Ro-BatCoV HKU9) in genome characteristics, it is sufficiently distinct to be classified as a new species according to the criteria defined by the International Committee of Taxonomy of Viruses (ICTV). More striking was that Ro-BatCoV GCCDC1 contained a unique gene integrated into the 3’-end of the genome that has no homologs in any known coronavirus, but which sequence and phylogeny analyses indicated most likely originated from the p10 gene of a bat orthoreovirus. Subgenomic mRNA and cellular-level observations demonstrated that the p10 gene is functional and induces the formation of cell syncytia. Therefore, here we report a putative heterologous inter-family recombination event between a single-stranded, positive-sense RNA virus and a double-stranded segmented RNA virus, providing insights into the fundamental mechanisms of viral evolution.

## Author Summary

Recombination is commonly reported in coronaviruses, and is an important mechanism by which these viruses generate genetic diversity. To date, however, most such recombination events involve homologous sequences among related viruses. We discovered a novel bat coronavirus that possesses a divergent but functional p10 gene that likely originated from, or shared the ancestry with, an ancestral non-enveloped orthoreovirus, thereby representing the outcome of heterologous recombination. We report herein a fusion-associated small transmembrane (FAST) protein encoded in an enveloped virus that arose through a putative inter-family recombination between a single-stranded, positive-sense RNA virus and a double-stranded segmented RNA virus. These findings shed important new light on the mechanisms of viral evolution and particularly the importance and scope of heterologous recombination.

## Introduction

Coronaviruses are large, enveloped viruses with single-stranded, positive-sense, non-segmented RNA genomes [1]. Based on the current nomenclature of the International Committee of Taxonomy of Viruses (ICTV), coronaviruses of the family *Coronaviridae* are now classified into four genera: *alpha-*, *beta-*, *gamma-* and *deltacoronavirus* [2, 3]. Betacoronaviruses can be further subdivided into four phylogenetic groups [2].

Coronaviruses employ a unique mechanism of viral genome replication and RNA synthesis, resulting in high frequencies of both mutation and recombination [4]. Recombination appears to be particularly important in coronavirus evolution [5], with a number of hotspots interspersed throughout the viral genome [6]. Recombination events at 3’-end of the genome might impact the replication ability of coronaviruses since there are a number of regulatory sequences and accessory genes in this region [5].

As coronaviruses were previously known to cause only mild respiratory illnesses in humans they were not a major concern of the public health community. However, the emergence of severe acute respiratory syndrome coronavirus (SARS-CoV) [7–9] and its high infectivity and fatality generated considerable interest in the biodiversity, genomics, evolution, natural hosts and potential inter-species transmission of coronaviruses [10]. To date, at least 90 types of coronavirus have been isolated or genome-identified from humans and a wide variety of animals, including domestic animals, wild birds and bats. Bats are particularly notable in this respect because they are known to harbor a diverse range of pathogens, and are known to be the reservoir hosts of both human coronavirus 229E [11] and SARS-CoV [12], and are closely related to MERS-CoV [13, 14]. As a consequence bats have been prioritized for the surveillance of emerging zoonotic diseases [15–17].

In the present study we report a novel coronavirus discovered from bat samples in China that has been tentatively named Rousettus bat coronavirus GCCDC1 (Ro-BatCoV GCCDC1). Multiple lines of evidence indicate that Ro-BatCoV GCCDC1 may have arisen from a recombination event between an ancestral coronavirus and a fusogenic orthoreovirus.

## Results

### A novel coronavirus with a putative recombinant reovirus gene

A total of 118 rectal swab samples from *Rousettus leschenaulti* bats sampled in Yunnan province China were screened for the presence of coronavirus RNA. Of these, 47 (40%) samples were found to be coronavirus positive. The PCR products were sequenced and BLAST searches revealed the sequences to be authentic coronavirus genes, with the strongest similarity to Rousettus bat coronavirus HKU9 (Ro-BatCoV HKU9) [18], a member of the genus *betacoronavirus* (group D). However, our attempts to isolate the virus from samples using a number of cell lines, including Vero E6, BHK-21, MDCK, A549, HEp-2, CaCo-2 and a bat cell line from Myotis kidney, were unsuccessful. The cell lines were inoculated with positive samples and three blind passages were performed for each sample. No cytopathic effect was observed in any passage, and there was an absence of viral replication from the culture supernatant and cell pellet of each passage.

**Fig 1:**
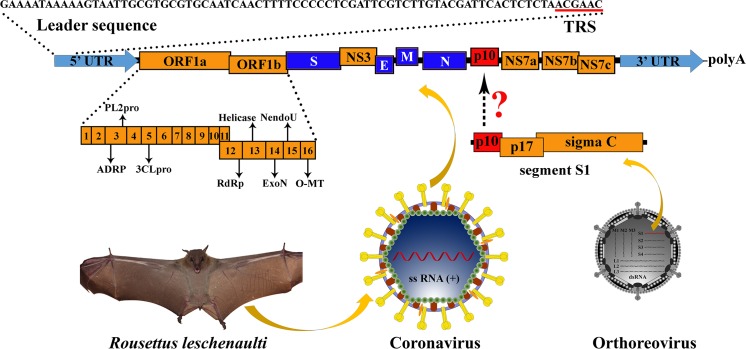
Genome organization and phylogenetic history of Ro-BatCoV GCCDC1. Genome organization of Ro-BatCoV GCCDC1. Nonstructural genes and putative mature nonstructural proteins, structural genes, and 5’- and 3’-UTR are illustrated with yellow, dark blue and light blue colors, respectively. The remarkable p10 gene is shown in red. The potential origin of the p10 gene is indicated by a dotted arrow and a question mark. The leader sequence and leader transcription regulatory sequence (TRS) are directly shown with nucleobases. The bat, *Rousettus leschenaulti*, is used to show the host species that Ro-BatCoV GCCDC1 was discovered. The schematic virion of coronavirus is used to show the virus that identified in the present study. The schematic virion of orthoreovirus and the segment S1 of the genome that it contains are used to demonstrate the possible origin of the p10 gene.

The viral genomic sequences present in two coronavirus positive samples (numbers 346 and 356) were determined with next-generation sequencing (NGS). Analysis using a partial (816-bp fragment) sequence of the RNA-dependent RNA polymerase (RdRp) gene indicated that the newly identified virus was likely to be a novel coronavirus according to previously proposed criteria [19]. Therefore, this virus was tentatively designated as Rousettus bat coronavirus GCCDC1 (Ro-BatCoV GCCDC1). Gaps within the genome of Ro-BatCoV GCCDC1 were closed, and the complete genome sequence confirmed, using Sanger sequencing. Finally, the 5’- and 3’-ends of Ro-BatCoV GCCDC1 genome were obtained using 5’ and 3’ RACE (Fig 1).

Excluding the polyadenylated tail at the 3’-terminus, the genome of Ro-BatCoV GCCDC1 was 30,129 nt in length with a G/C content of 45.4%. Comparative genomic sequence analysis indicated that Ro-BatCoV GCCDC1 was most closely related to Ro-BatCoV HKU9 strains [18] with 66.6% - 67.4% nucleotide identities. Similarly, Ro-BatCoV GCCDC1 displayed equivalent genomic characteristics to Ro-BatCoV HKU9 except for an inserted gene at 3’ end (discussed in detail in the next section). The major open reading frames (ORFs) had the identical order, namely 5’- replicase ORF1ab—spike (S)—NS3—envelope (E)—membrane (M)—nucleocapsid (N) followed by the accessory genes encoding nonstructural proteins (NSPs) (Fig 1 and Table 1), although the N gene was truncated. Amino acid sequence analyses showed that the ORF1ab, S, NS3, E, M and N proteins of Ro-BatCoV GCCDC1 shared higher identities with Ro-BatCoV HKU9 strains than those of other betacoronaviruses (Table 1). Also of note was that the 3’-end of Ro-BatCoV GCCDC1 genome, just downstream of N gene, possessed a much more complicated structure than those of other members in the genus. Clearly, there were four NSP-encoding ORFs. According to the convention, the second-to-fourth ORFs were temporarily named NS7a, NS7b and NS7c, respectively, since they shared 29% - 53% amino acid identities with the accessory genes of Ro-BatCoV HKU9 strains and other related bat coronaviruses (S1 Table). Perhaps the most striking feature of Ro-BatCoV GCCDC1 genome was the presence of a small intact ORF with 276 bases embedded between the N and NS7a genes. Although this ORF that had no homology to any known coronavirus, the encoded protein exhibited 30% - 54.9% amino acid identity with the p10 protein encoded by the first ORF of segment S1 of avian and bat fusogenic orthoreoviruses [20], which are double-stranded segmented RNA viruses belonging to the family *Reoviridae*. Therefore, this ORF was provisionally marked as p10 according to the molar weight of protein that it encodes (Fig 1).

**Table 1:** Coding potential, transcription regulatory sequences and sequence comparisons of Ro-BatCoV GCCDC1 with Ro-BatCoV HKU9 strains, SARS-CoV, BatCoV HKU3 stains, MERS-CoV, BatCoV HKU4 strains and BatCoV HKU5 strains.

ORFs	Nucleotide positions (start to end)	Predicted size (aa) of protein	Pairwise amino acid identity (%)^a^ Ro-BatCoV GCCDC1 *vs*						Leader TRS region and intergenic TRS^b^	Distance (nt) from TRS to ATG
			HKU9s^c^	SARS-CoV^d^	HKU3s^e^	MERS-CoV^f^	HKU4s^g^	HKU5s^h^		
**ORF 1ab**	235–21155	6973	74.6–75.4	47.1	47.3–47.4	46.3	45.7–45.8	46.1–46.2	TCTA**ACGAAC**TTAA	156
**Spike**	21121–24993	1290	58.8–66.4	32.1	31.3–31.4	27.9	28.4–28.6	27.7–27.8	CTGA**ACGAAC**TAGT	42
**NS3^i^**	24990–25676	228	48.9–50.7	*NA*	*NA*	*NA*	*NA*	*NA*	GAAA**ACGAAC**GGCA	3
**E**	25676–25906	76	61.8–69.7	32.9	32.9	23.7	26.3	26.3	GATG**TCGAAC**TGTG	4
**M**	25914–26579	221	75.6–80.5	43.2	42.3–42.7	41.1	41.1	43.6	TCTA**ACGAAC**AGGA	27
**N**	26627–27958	443	68.0–71.7	39.1	39.5–40.0	41.7	41.4–41.7	38.4–38.7	GGAA**ACGAAC**CTAT	5
**p10^j^**	27994–29269	91	*NA*	*NA*	*NA*	*NA*	*NA*	*NA*	AATG**ACAAAC**CCAA	97
**NS7a ^k^**	28287–28850	187	40.0	*NA*	*NA*	*NA*	*NA*	*NA*	TCTA**ACGAAC**TCAT	5
**NS7b ^l^**	28863–29444	193	32.0–42.0	*NA*	*NA*	*NA*	*NA*	*NA*	TCTA**ACGAAC**TATG	1
**NS7c^m^**	29444–29893	149	30.0–53.0	*NA*	*NA*	*NA*	*NA*	*NA*	GTAG**ACGAAC**TTTG	4

**a:** Calculated with MEGA6 [21] using a pairwise deletion option [13].

**b:** Underlined and bold type indicates the conserved nucleotide in the TRS core sequence.

**c:** GenBank accession numbers of the viruses used in this analysis: NC_009021, EF065514, EF065515, EF065516, HM211098, HM211099, HM211100, HM211101.

**d:** GenBank accession numbers of the viruses used in this analysis: NC_004718.

**e:** GenBank accession numbers of the viruses used in this analysis: DQ022305, DQ084199, DQ084200, GQ153539, GQ153540, GQ153541, GQ153542, GQ153543, GQ153544, GQ153545, GQ153546, GQ153547, GQ153548.

**f:** GenBank accession numbers of the viruses used in this analysis: NC_019843.

**g:** GenBank accession numbers of the viruses used in this analysis: NC_009019, EF065506, EF065507, EF065508.

**h:** GenBank accession numbers of the viruses used in this analysis: NC_009020, EF065510, EF065511, EF065512.

**i:** In the genomes of Ro-BatCoV GCCDC1, Ro-BatCoV HKU9 strains, SARS-CoV, BatCoV HKU3 strains, MERS-CoV, BatCoV HKU4 strains and BatCoV HKU5 strains, the organization of the region between the spike and E genes is divergent; there is no comparability of ORFs in this region.

**j:** As the p10 gene is specific to Ro-BatCoV GCCDC1, there are no homologous genes in the genomes of Ro-BatCoV HKU9, SARS-CoV, BatCoV HKU3, MERS-CoV, BatCoV HKU4 or BatCoV HKU5.

**k:** The NS7a protein of Ro-BatCoV GCCDC1 shares 40% amino acid identity (87% coverage) with the NS7a protein of Bat coronavirus HKU9-1; There is no comparability with any ORF of other coronaviruses.

**l:** The NS7b protein of Ro-BatCoV GCCDC1 shares 32% - 42% amino acid identity (88% - 91% coverage) with the NS7a proteins of Bat coronavirus HKU9-3, HKU9-4, HKU9-10-1 and HKU9-10-2.

**m:** The NS7c protein of Ro-BatCoV GCCDC1 shares 30% - 53% amino acid identity (90% - 99% coverage) with the NS7b protein of Bat coronavirus HKU9-1, HKU9-2, HKU9-3, HKU9-4, HKU9-10-1 and HKU9-10-2.

The putative leader and body transcription regulatory sequences (TRSs) of Ro-BatCoV GCCDC1, and their genomic localizations, were predicted in accordance with consensus core sequences of the TRSs of betacoronaviruses (Table 1). The TRS core sequence, 5’-ACGAAC-3’, was consistent with those of SARS-CoV, Ro-BatCoV HKU9 and other betacoronaviruses. From the location of leader TRS, the leader sequence of the genome was then identified, which spanned genome positions 1 (G) to 78 (C) (Fig 1 and Table 1). Notably, in the putative TRS of the p10 gene, there was one nucleobase difference with the consensus core sequence (Table 1).

The putative mature nonstructural proteins (NSPs) in the ORF1ab encoding the replicase were calculated based on the cleavage and recognition pattern of the 3C-like proteinase (3CLpro) and papain-like proteinase (PLpro). Comprehensive information on the size and genomic locations of nsp1 to nsp16 and the putative cleavage sites of proteinases is presented in Table 2. Previous studies indicated that the P1 position of 3CLpro specific cleavage site is exclusively occupied by a glutamine (Q) residue [22, 23]. However, nucleobase 12642 in the Ro-BatCoV GCCDC1 genome was a T nucleotide, thereby changing glutamine (Q) to histidine (H). More interestingly, there were no glutamine codons in the sequence (from -273 to +192) around this site, as also observed in the corresponding site in the genomes of Ro-BatCoV HKU9. Therefore, the LH|AG region may represent a potential alternative cleavage site of 3CLpro to cleave between NSP9 and NSP10. A similar phenomenon may occur at the cleavage site between NSP10 and NSP12 of Ro-BatCoV GCCDC1, where the CAG codon has mutated to CAC causing the conversion of Q to H in amino acid sequence (Table 2).

**Table 2:** Prediction of the putative pp1a/pp1ab cleavage sites of Ro-BatCoV GCCDC1 based on comparison with prototype coronaviruses^a^.

NSP	amino acids positions in ORF1a/ORF1b	Predicted size (aa) of protein	C-end putative cleavage site	Putative functional domain(s)^b^
**NSP1**	M^1^-G^174^	174	RG|GN	Unknown
**NSP2**	G^175^-G^772^	598	GG|GK	Unknown
**NSP3**	G^773^-G^2653^	1881	VG|GN	ADRP, PL2 pro
**NSP4**	G^2654^-Q^3147^	494	LQ|AG	Hydrophobic domain
**NSP5**	A^3148^-Q^3453^	306	LQ|SR	3CL pro
**NSP6**	S^3454^-Q^3741^	288	IQ|SN	Hydrophobic domain
**NSP7**	S^3742^-Q^3824^	83	LQ|AV	Unknown
**NSP8**	A^3825^-Q^4024^	200	LQ|NN	Primase
**NSP9**	N^4025^-H^4136^	112	**LH|AG**	Unknown
**NSP10**	A^4137^-H^4275^	139	**LH|AN**	Unknown
**NSP11**	A^4276^-S^4289^	14		Unknown (short peptide at the end of ORF1a)
**NSP12**	A^4276^-Q^5207^	932	LQ|SV	RdRp
**NSP13**	S^5207^-Q^5808^	601	TQ|SA	HEL, NTPase
**NSP14**	S^5809^-Q^6338^	530	LQ|SL	ExoN, NMT
**NSP15**	S^6339^-Q^6680^	342	LQ|SK	NendoU
**NSP16**	S^6681^-V^6973^	293		O-MT

a: GenBank accession numbers of the viruses used in this analysis: SARS-CoV, NC_004718; HCoV HKU1, NC_006577; IBV, NC_001451; TCoV, NC_010800; BCoV, NC_003045; MHV, NC_001846; and PEDV, NC_003436.

b: ADRP, ADP-ribose 1-phosphatase; PL2pro, papain-like protease 2; 3CL pro, coronavirus nsp5 protease; HEL, helicase; NTPase, nucleoside triphosphatase; ExoN, exoribonuclease; NMT, N7 methyltransferase; NendoU, endoribonuclease; OMT, 2’-O-methyltransferase.

Following the criteria for coronavirus species demarcation defined by the ICTV [1, 13], seven conserved replicase domains of Ro-BatCoV GCCDC1 were selected for analysis (Table 3). The amino acid identities of seven concatenated domains in Ro-BatCoV GCCDC1 revealed that they shared 84.4% - 84.8% identity with those of Ro-BatCoV HKU9, which was below the 90% threshold used for species demarcation (Table 3). Hence, these data suggest that the newly identified Ro-BatCoV GCCDC1 represents a novel coronavirus species in the genus *betacoronavirus*.

**Table 3:** Comparison of amino acid identities of seven conserved replicase domains of Ro-BatCoV GCCDC1 for species classification.

Coronavirus CCDC1 strain 356	amino acid identity (%)^a^					
	HKU9s^b^	SARS-CoV^c^	HKU3s^d^	MERS-CoV^e^	HKU4s^f^	HKU5s^g^
**ADRP**	65.8–68.3	48.8	49.4–50.0	45.0	42.5–43.1	39.4
**NSP5 (3CL pro)**	83.3–84.3	52.3	52.0–52.3	49.2	49.8	49.5–49.8
**NSP12 (RdRp)**	89.8–90.3	72.3	72.3–72.4	69.7	70.1–70.2	70.5–70.6
**NSP13 (Hel, NTPase)**	90.8–91.5	73.7	73.7–74.0	72.7	73.1	72.9–73.1
**NSP14 (ExoN, NMT)**	82.1–82.8	61.4	61.8–62.1	61.1	60.1	60.7
**NSP15 (NendoU)**	70.9–73.3	49.7	49.4–49.7	46.6	46.0	47.5–47.8
**NSP16 (OMT)**	80.1–84.2	63.8	63.1–63.5	61.8	62.1	62.5
**Concatenated domains**	84.4–84.8	64.4	64.4–64.6	62.4	62.3–62.4	62.5–62.6

**a:** Calculated with MEGA6 [21] using a pairwise deletion option [13].

**b:** GenBank accession numbers: NC_009021, EF065514, EF065515, EF065516, HM211098, HM211099, HM211100, HM211101.

**c:** GenBank accession number: NC_004718.

**d:** GenBank accession numbers: DQ022305, DQ084199, DQ084200, GQ153539, GQ153540, GQ153541, GQ153542, GQ153543, GQ153544, GQ153545, GQ153546, GQ153547, GQ153548.

**e:** GenBank accession numbers: NC_019843.

**f:** GenBank accession numbers: NC_009019, EF065506, EF065507, EF065508.

**g:** GenBank accession numbers: NC_009020, EF065510, EF065511, EF065512.

**Fig 2:**
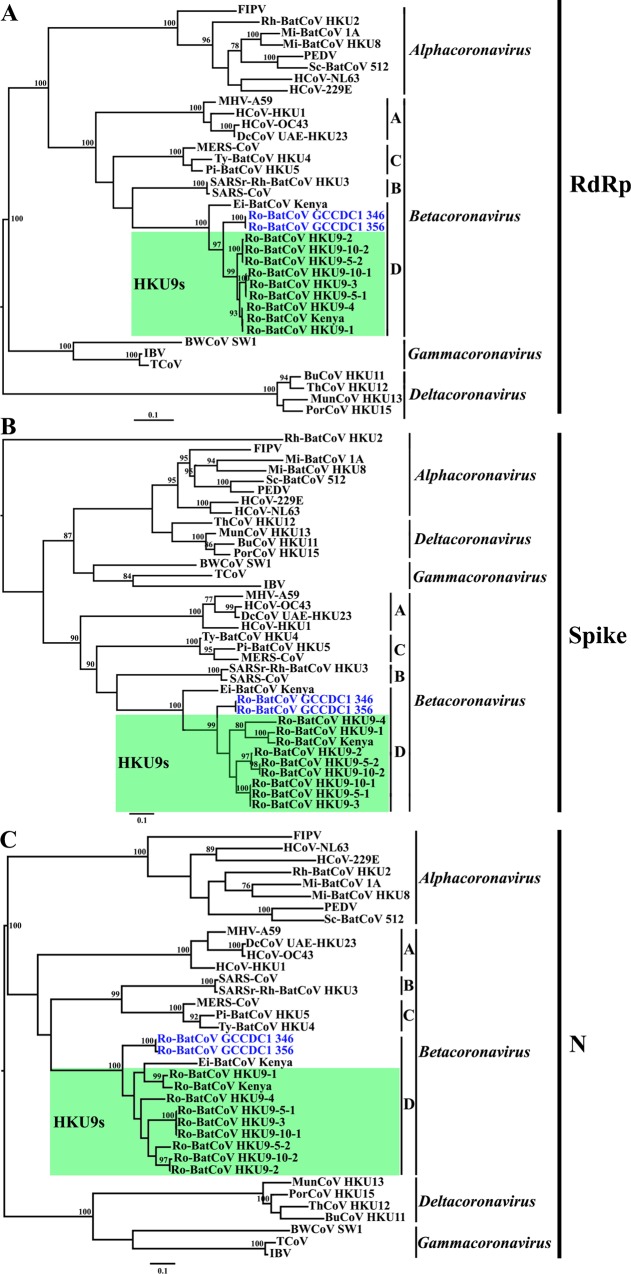
Phylogenetic analyses of representative coronaviruses, including Ro-BatCoV GCCDC1. All trees (A: RdRp; B: S and C: N) were inferred using the maximum likelihood method available in PhyML. Bootstrap values are shown at relevant nodes. The GenBank accession numbers used in this analysis are listed in S2 Table.

**Fig 3:**
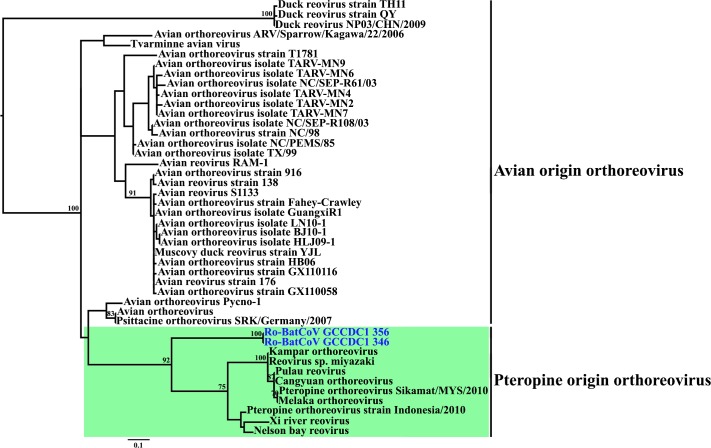
Phylogenetic analyses of p10 from representative reoviruses and Ro-BatCoV GCCDC1. The tree was inferred using the maximum likelihood method available in PhyML. Bootstrap values are shown at relevant nodes. The GenBank accession numbers used in this analysis are listed in S3 Table.

To determine the evolutionary position of Ro-BatCoV GCCDC1, the RdRp, S, N and p10 proteins were subjected to phylogenetic analyses. Phylogenetic trees of the RdRp, S and N proteins illustrated that Ro-BatCoV GCCDC1, Eidolon bat coronavirus/Kenya/KY24/2006 (Ei-BatCoV Kenya), Rousettus bat coronavirus/Kenya/KY06/2006 (Ro-BatCoV Kenya) and Ro-BatCoV HKU9 strains all belong to group D of the genus *betacoronavirus* (Fig 2). Within this cluster the two Ro-BatCoV GCCDC1 strains (346 and 356) formed a distinct lineage that was a sister-group to Ro-BatCoV Kenya and the Ro-BatCoV HKU9 strains (maximum bootstrap value of 100%). However, a strikingly different phylogenetic pattern was observed in the distinctive p10 protein (Fig 3), in which the Ro-BatCoV GCCDC1 sequences were clearly related to bat (Pteropine) originated orthoreoviruses. Although the branch leading to the Ro-BatCoV GCCDC1 sequences is long, these viruses are clearly more closely related to the bat-origin than avian-origin orthoreoviruses (Fig 3), matching the host species from which Ro-BatCoV GCCDC1 was isolated.

### Evidence for heterologous recombination of a reovirus p10 gene recombination into Ro-BatCoV GCCDC1

**Fig 4:**
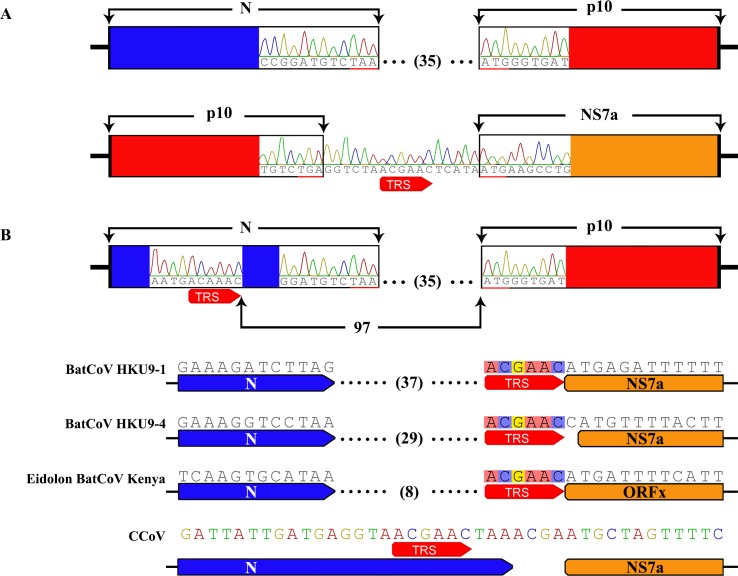
Identification of the recombinant p10 gene and its TRS. (A) Confirmation of the “exotic” p10 gene. The sequences that cover the upstream junction site between the N and p10 genes, and downstream junction site between the p10 and NS7a genes, are illustrated with sequencing patterns. The length of the intergenic sequence between the N and p10 genes is indicated with a number. The TRS preceding the NS7a gene in the intergenic sequence is marked with red arrow. (B) Identification of the TRS of the p10 gene. The TRS of the p10 gene in the N gene is illustrated with a sequencing pattern. The distance from the TRS to the AUG codon of p10 gene is indicated with a number. The length of the intergenic sequence between the N gene and genes just downstream of N gene are indicated with numbers. The TRSs of genes just downstream of N gene are marked with red arrows.

To exclude the false amplification of DNA polymerase or the inaccurate assembly of NGS data, the NGS data was analyzed further. Read mapping determined that there were a set of reads that covered the upstream junction site (i.e. the recombination break-point) between N and p10 genes, and a downstream junction site between the p10 and NS7a genes (S1 Fig with data in S1 File). In addition, the integrity and continuity of context sequence surrounding the p10 gene were confirmed with specific primers. Agarose gel electrophoresis showed that the PCR products were intact fragments of the expected length. The amplicons were cloned for sequencing. As shown in Fig 4A, the sequence obtained covers, without interruption, the partial N gene, the whole p10 gene and partial NS7a gene (data in the S2 File). Hence, there is clear evidence that the recombination event that placed the reovirus p10 gene in the Ro-BatCoV GCCDC1 genome was genuine.

**Fig 5:**
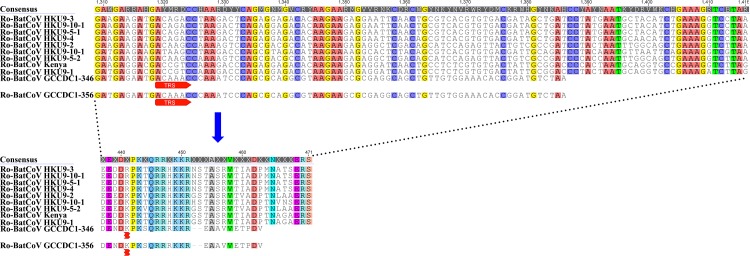
Comparison of the 3'-terminus of the N gene of Ro-BatCoV GCCDC1 with those of Ro-BatCoV HKU9 strains and Ro-BatCoV Kenya. Alignment of nucleotide and amino acid sequence of the 3'-terminus of the N gene among Ro-BatCoV GCCDC1, Ro-BatCoV HKU9 strains and Ro-BatCoV Kenya. The eight amino acid truncation and two-amino-acid deletion at the 3’-terminus of N protein of the Ro-BatCoV GCCDC1 are illustrated.

Sequencing information confirmed that the TRS of p10 gene is located within the encoding sequence of N gene with the core sequence of 5’-ACAAAC-3’, which exhibited a single nucleobase difference to the consensus core sequence (5’-ACGAAC-3’) (Fig 4B). We also observed a 97 nucleobase sequence between the TRS and the p10 initiation codon (Fig 4B), which was much longer than the intervening sequences of other genes with the exception for that between the leader TRS and ORF1ab (Table 1). As shown in Fig 4B and S2 Fig, sequence comparisons also revealed that the location of TRS in the p10 gene could be discriminated from those of other genes, which are adjacent and downstream to the N gene of Group D *Betacoronavirus*. Notably, the ORF of the Ro-BatCoV GCCDC1 N gene was disrupted by the insertion of the “exotic” p10 gene, causing the truncation of eight amino acids at the 3’-terminus and a two amino acid deletion (Fig 5).

### Subgenomic structures of Ro-BatCoV GCCDC1

**Fig 6:**
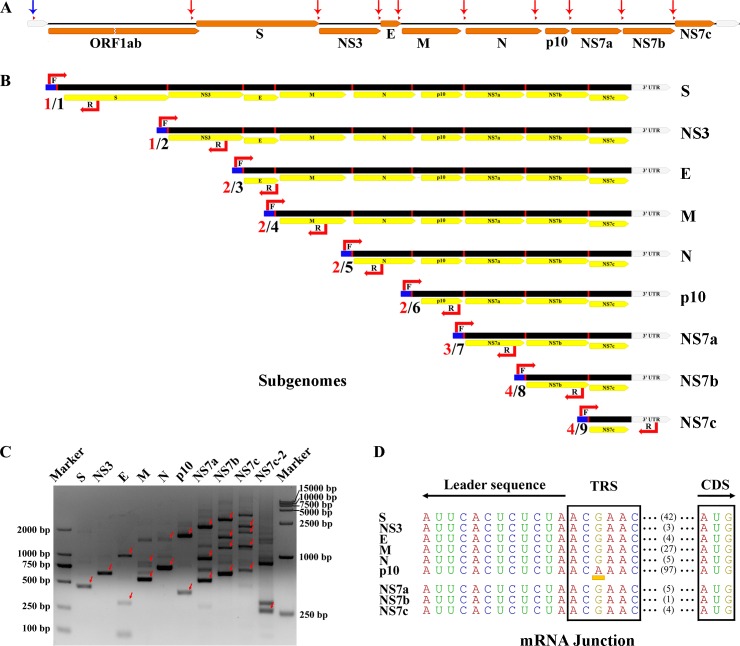
Subgenomic structures of Ro-BatCoV GCCDC1. (A) Schematic of the Ro-BatCoV GCCDC1 genome. The genome is represented by a black line; ORFs, and the 5’-UTR and 3’-UTRs are indicated by yellow and grey arrows, respectively. The TRSs are marked with small red triangles. The genomic locations of the leader and body TRS(s) are shown with blue and red arrows, respectively. (B) Schematic structures of putative transcribed subgenomic mRNAs. Subgenomes are represented by a black rectangles and the common leader sequence is denoted by a blue box. The target sites of forward and reverse primers are marked and indicated with letter F and R, respectively. Two numbers are shown in front of each subgenomic mRNA. The black number to the right of the slash indicates the potential number of fragment(s) that could be amplified using this set of primers, while the red one to the left represents the actual numbers of the fragment(s) obtained in this experiment which corresponds to the number of band(s) on each lane marked with a red arrow(s) on the agarose gel. (C) Agarose gel electrophoresis of the PCR products of subgenomic mRNA. The lowest band marked with a red arrow on each lane is the specific amplicon of each subgenomic mRNA. Other marked bands are amplicons of upper subgenomic mRNAs as shown in Fig 6B. (D) mRNA junctions of the detected subgenomic mRNAs. The TRSs and fusion sites are shown in a black frame. The bias of the TRS of p10 gene is highlighted with a yellow block. The leader sequence and CDS are indicated. The lengths of intergenic sequences are shown with numbers.

According to the information provided above, the relative locations of the putative leader and body TRS(s) were identified in the genome of Ro-batCoV GCCDC1 (Fig 6A). Based on the TRSs and transcription mechanism of coronavirus, nine potential subgenomic mRNAs of Ro-BatCoV GCCDC1, including S, NS3, E, M, N, p10, NS7a, NS7b and NS7c, were depicted (Fig 6B). In addition to an identical 5’ leader sequence, each lower subgenomic mRNA shared the same 3’-end structure with the upper one to comprise a 3' co-terminal nested set with the genome.

The presence of subgenomic mRNAs is strong evidence of coronavirus replication in the infected cells. To determine if the bat, which sample was collected from, was likely the natural host of Ro-BatCoV GCCDC1, subgenomic mRNAs in the sample were probed with a comprehensive set of primers. The PCR products were confirmed on an agarose gel. As displayed in Fig 6C, the lowest band marked with a red arrow on each lane was the specific amplicon from each subgenomic mRNA as demonstrated in Fig 6B. However, additional amplified bands were also compatible with this inference. As shown as an example on the lane of the E gene, the upper band indicates that subgenomic mRNA NS3 was simultaneously amplified in this reaction. On each lane the lowest band was cloned for sequencing, while other bands were purified and sequenced directly. Since the specific amplicon of the subgenomic mRNA NS7c failed to be cloned into the vectors, the PCR product was used as template for a second round of nested PCR. The product was then confirmed as shown in the lane of NS7c-2 in Fig 6C, and the band was cloned for sequencing. The results (Fig 6D and S3 Fig) indicated that the core sequence of the leader and body TRS of each gene, the leader-body fusion sites, and the mode of generation of subgenomic mRNAs were consistent with the prediction and demonstration in Fig 6B, especially the p10 gene and its subgenomic mRNA. Therefore, the existence of subgenomic mRNA in the samples further proved that the p10 gene was an intact authentic gene in the genome of Ro-BatCoV GCCDC1.

### The p10 gene of Ro-BatCoV GCCDC1 is functional

**Fig 7:**
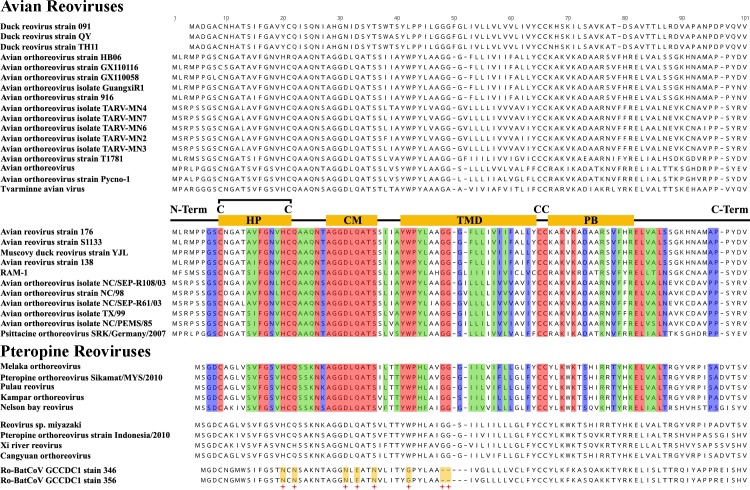
Comparison of the p10 protein of Ro-BatCoV GCCDC1 with those of avian and bat origin orthoreovirus. The absolutely, highly, moderately and non-conserved amino acids of p10 proteins as defined previously [26], are illustrated with red, blue, green and black colors, respectively. The motifs and domains in the p10 molecule are represented as previously reported [26]. Motifs present in the ectodomain (HP, hydrophobic patch; CM, conserved motif), endodomain (PB, polybasic) and the central transmembrane domain (TMD) are depicted with yellow rectangles. The four conserved cysteine residues (C) are shown. The two cysteines in the ectodomain form an intra-molecular disulfide bond. Comparison of the p10 protein of Ro-BatCoV GCCDC1 with those of avian and bat origin orthoreoviruses, the 8 different amino acids (including a 2 amino acid deletion) in the 28 absolutely conserved amino acids are symbolized with red star.

**Fig 8:**
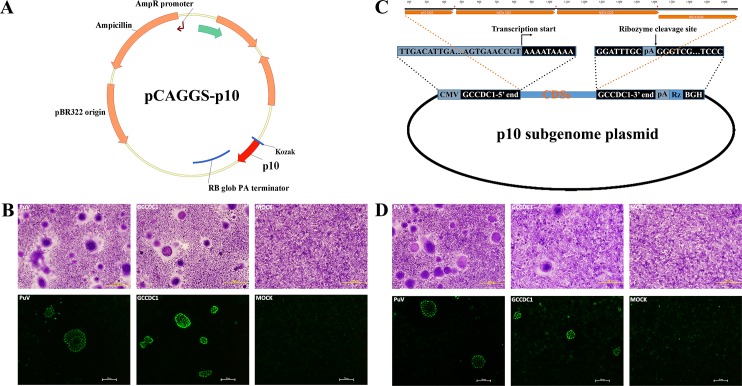
Syncytium formation and functional analyses of Ro-BatCoV GCCDC1 p10 gene. (A) The construction of transient expression plasmid of p10 gene based on a pCAGGS vector. (B) Transient expression of the p10 gene and syncytium formation. **Top:** the observation of syncytium formation with Wright-Giemsa staining on the monolayer BHK-21 cells transfected with recombinant plasmid of Pulau virus p10 gene, recombinant plasmid of Ro-BatCoV GCCDC1 p10 gene, and empty pCAGGS vector; **Bottom:** the observation of syncytium formation with indirect immunofluorescence staining on the cells treated as described above. (C) The construction of subgenomic plasmid of p10 gene. The putative subgenome of p10 was cloned into a pcDNA3.0-derived vector. (D) Transient expression of the p10 gene and syncytium formation with recombinant subgenomic p10 plasmid. **Top:** the observation of syncytium formation with Wright-Giemsa staining on the monolayer BHK-21 cells transfected with recombinant plasmid of Pulau virus p10 gene, recombinant plasmid of p10 subgenome of Ro-BatCoV GCCDC1 and empty pcDNA3.0 vector; **Bottom:** the observation of syncytium formation with indirect immunofluorescence staining on the cells treated as described above. (Wright-Giemsa staining: stained monolayers were imaged using an Olympus IX51FL+DP70 microscope under 100× magnification, scale bars = 200 μm; indirect immunofluorescence staining: stained monolayers were imaged using a Nikon DIAPHOT-TMD microscope under 200× magnification, scale bars = 50 μm).

Despite the orthoreovirus origin of p10, this protein exhibited 8 amino acid differences (including a 2 amino acid deletion) among the 28 “absolutely conserved” amino acids described previously (Fig 7). Hence, it is necessary to investigate whether the p10 gene of Ro-BatCoV GCCDC1 could play the same role as its reovirus homologs. For this purpose, the p10 gene of Ro-BatCoV GCCDC1 was transiently expressed in BHK-21 cells as well as the p10 gene of Pulau virus, which was used as a positive control. Wright-Giemsa and immunofluorescence staining showed that both genes had the same function to induce the formation of cell syncytia (Fig 8A and 8B and S4A Fig). Thus, the alteration of certain conserved amino acids did not impair the syncytiogenesis of p10 gene of Ro-BatCoV GCCDC1.

The p10 subgenomic mRNA identified in the samples confirmed that the p10 gene could be transcribed from the genome of Ro-BatCoV GCCDC1. However, due to the failure of virus isolation (despite a great effort), there is no effective way to judge whether the p10 gene could be expressed during the virus replication cycle. Therefore, an artificial plasmid was constructed containing the transcribed p10 subgenomic mRNA, which confirmed the functional expression of p10 (Fig 8C). When the plasmid was transfected into BHK-21 cells, once again, cell syncytia were observed with Wright-Giemsa and immunofluorescence staining (Fig 8D and S4B Fig). Thus, this indirect evidence suggests that the p10 gene functions during the replication cycle of Ro-BatCoV GCCDC1.

Immunofluorescence staining also showed that polyclonal antibodies of Ro-BatCoV GCCDC1 p10 protein reacted with the p10 protein of Pulau virus (Fig 8B and Fig 8D). In addition, the cross-reactivity further proved that the p10 gene of Ro-BatCoV GCCDC1 might have the same origin as those of fusogenic orthoreoviruses.

As the p10 protein of Ro-BatCoV GCCDC1 is the first report of FAST protein in an enveloped virus, the conserved amino acids of the p10 protein of Ro-BatCoV GCCDC1 were mutated to determine whether they play a vital role in cell-to-cell fusion and syncytium formation as those sites in the p10 protein of reoviruses (S5A Fig) [24, 25]. Notably, no cell syncytia were observed for all mutant constructs of p10 which had substitutions in the previously defined key sites in the p10 protein of reoviruses (S5B Fig). This indicates that the functionality of p10 in Ro-BatCoV GCCDC1 also depends on traditional conserved domains relevant for the function of the FAST protein [24, 25].

To confirm the expression of p10 by the Ro-BatCoV GCCDC1 virus, we performed Western blotting (WB) to detect the presence of p10 protein in the bat feces and concentrated rectal swab specimens. The results revealed the expression of p10 by Ro-BatCoV GCCDC1 itself (S6 Fig).

## Discussion

We have identified a novel coronavirus, Ro-BatCoV GCCDC1, from *Rousettus leschenaulti*, that belongs to group D of the genus *betacoronavirus* and which is related to Ro-BatCoV HKU9 [18]. According to the criteria defined by ICTV [1], Ro-BatCoV GCCDC1 is sufficiently divergent to represent a novel bat coronavirus. More striking was that Ro-BatCoV GCCDC1 contains a p10 protein located at the 3’-end of the genome that appears to have captured from a bat-origin orthoreovirus by heterologous recombination.

Homologous recombination events frequently occur during the viral RNA replication of coronaviruses, and are important for their evolution [10, 27–29]. However, it is also possible that coronaviruses are one of the few virus families that can experience heterologous recombination. For example, members of *betacoronavirus* group A possess an HE gene [30, 31] which was seemingly derived from ancestral influenza C virus, a negative-stranded RNA virus with a segmented genome [30, 31], and which would represent another case of inter-family recombination, although it has also been proposed that the HE gene might be captured from host RNA [32]. Uncommon inter-family recombination events have also been reported in chicken infectious anemia virus [33], bandicoot papillomatosis carcinomatosis virus type 1 [34], and recombinant viruses between Marek’s disease virus, fowlpox virus, and various avian retroviruses [35, 36]. In the current study, sequence, phylogenetic and functional analyses demonstrated that the p10 gene of Ro-BatCoV GCCDC1 was likely derived from an ancestral orthoreovirus, although that it occupies a divergent position in the phylogeny suggests that the direct ancestor of the recombination event has yet to be sampled. Hence, these data provide clear evidence for a putative inter-family recombination between a single-stranded, positive-sense RNA virus and a double-stranded segmented RNA virus. The mechanisms that underpin such inter-family heterologous recombination clear merit further investigation.

The biggest difference between fusogenic and nonfusogenic orthoreoviruses is the presence/absence of a small protein encoded by the segment S1 of the genome, termed the fusion-associated small transmembrane (FAST) protein. The FAST proteins are the only known nonenveloped reovirus fusogens that can mediate cell-to-cell, but not virus-cell, membrane fusion to induce the formation of syncytia [20], and which might promote the dissemination of virus among cells [37]. Thus, the FAST proteins are the pathogenic determinants of fusogenic orthoreoviruses. To date, the FAST family comprises six members including p10 proteins encoded by avian- and bat-origin orthoreoviruses, p13, p14 and p15 encoded by broome virus, reptilian reovirus and bush viper reovirus and baboon orthoreovirus respectively, and p16 and p22 encoded by aquareoviruses. Intriguingly, a specific p10 gene was identified in Ro-BatCoV GCCDC1, so that this is the first report of a FAST protein in an enveloped virus and hence could represent the seventh member of FAST family.

Unfortunately, the isolation of Ro-BatCoV GCCDC1 failed on cell culture in the present study, so it is difficult to determine the role of p10 gene during the life cycle of Ro-BatCoV GCCDC1. However, functional analysis showed that the coronavirus p10 gene could induce syncytium formation in the transfected cells, in the same manner as orthoreoviruses, which might be beneficial for cell-to-cell virus spread. It is therefore possible that the p10 protein enhances the transmission potential of Ro-BatCoV GCCDC1. Previous studies of the potential recombination between coronavirus and influenza C virus revealed the pivotal role of the shared HE gene for the pathogenesis of *betacoronavirus* group A [38]. Interestingly, human coronavirus HKU1, OC43 and bovine coronaviruses employ the HE protein to mediate receptor-destroying enzyme activity late in the infection cycle to facilitate viral progeny release and achieve efficient virus dissemination [39].

Compared to nonfusogenic orthoreoviurses, fusogenic orthoreoviruses can cause severe pneumonia when infecting humans [40, 41], further implying that p10 is an important pathogenic determinant. Thus, the recombination of the reovirus-originated p10 into the Ro-BatCoV GCCDC1 may enable the novel virus to disseminate and replicate rapidly in the host, in turn leading to severe infections. In recent years, several coronaviruses, notably SARS-CoV and MERS-CoV, have caused severe pneumonia among humans [42, 43]. Because of the presence of human infected fusogenic orthoreoviruses such as Melaka virus (MelV) [44], there is obviously some risk that cross-family recombination events such as that described here may generate a novel coronavirus with altered pathogenicity. Our study therefore highlights the importance of investigating the mechanisms that might enable possible recombination between human coronaviruses and orthoreoviruses.

In the protein sequence of ORF1ab encoded replicase of Ro-BatCoV GCCDC1, the regular P1 position at two 3CLpro cleavage sites, NSP9/NSP10 and NSP10/NSP12, contains a Q to H mutation which may impair the proteolytic efficacy and the release of NSP9, NSP10 and NSP12. As NSP12 is a typical RNA polymerase and the core of replication-transcription complexes (RTC) and NSP10 usually serves as a molecular switch that can interact with multiple NSPs to form complexes, the replication ability of Ro-BatCoV GCCDC1 might be suppressed by the decrease of release of these vital elements. It is also interesting to note that a similar situation may be observed at the NSP13/NSP14 cleavage sites of replicase polyprotein of human coronavirus HKU1 and human coronavirus NL63. Clearly, further investigation will need to focus on the isolation of the virus, construction of infectious clones, and the virulence and replication ability of Ro-BatCoV GCCDC1 influenced by knockout of p10 gene and/or reverse mutation of cleavage sites.

Phylogenetically distinct virus species or lineages have been reported co-circulating in certain bat populations [45, 46]. Under this situation, co-infections of single host cells—the necessary requisite for recombination—are possible. By careful sequence analysis we show that the heterologous recombination event placed the p10 gene in Ro-BatCoV GCCDC1 is genuine. Previous studies showed the existence of p10-harboring orthoreoviruses in bat populations [40, 47–49], such that co-infections with bat coronaviruses and hence recombination events are clearly possible. In addition, a previous study reported that mammalian orthoreovirus, a type of nonfusogenic orthoreovirus, was isolated from a SARS-CoV patient along during the in 2003 outbreak [50]. We believe that future studies should investigate co-infections in specific bat cell lines using a coronavirus similar to Ro-BatCoV GCCDC1 or Ro-BatCoV HKU9 and the relevant orthoreoviruses, from which it will be possible to reveal more of the underlying basis of heterologous coronavirus recombination.

## Materials and Methods

### Ethics statement

The protocol in this study was approved by the Committee on the Ethics of Animal Care and Use of the Chinese Center for Disease Control and Prevention (Permit 20140509015). The study was conducted in accordance with the Guide for the Care and Use of Wild Mammals in Research of the People's Republic of China.

### Cell cultures

African green monkey kidney cells (Vero E6), human epithelial colorectal adenocarcinoma cells (CaCo-2), human epithelial type 2 HeLa derivative cells (HEp-2) and human lung carcinoma cells (A549) were purchased from the China Center for Type Culture Collection, while baby hamster kidney (BHK-21) and Madin-Darby canine kidney (MDCK) cells were obtained from the Cell Resource Center of the Shanghai Institute for Biological Sciences, Chinese Academy of Sciences. The immortalized kidney cell line of *Myotis Davidii* (IKMD) was a generous gift of Dr. Zhengli Shi, Wuhan Institute of Virology, Chinese Academy of Sciences. Cells were grown in Eagle’s minimum essential medium (EMEM) (A549, BHK-21) or in Dulbecco’s modified EMEM (DMEM) (Vero E6, CaCo-2, HEp-2, MDCK, IBIE and IKMD) supplemented with 10% or 20% (CaCo-2) fetal bovine serum in a humidified chamber containing 5% CO_2_ at 37°C.

### Sample collection

All the bats analyzed here were captured at a roosting site with the assistance of villagers and staff of local the CDC office in Xishuangbanna, Yunnan Province, China. The rectal swab samples were collected and placed in the cryotube with viral transport medium (VTM) containing Earle's balanced salt solution (Invitrogen, United States), 5% bovine albumin, 50,000 μg/ml vancomycin, 50,000 μg/ml amikacin, 10,000 units/ml nystatin [51]. All samples were immediately stored in liquid nitrogen and then transported with dry ice to our laboratory in Beijing and stored in the ultra-low temperature freezer until used for RNA extraction.

### RNA extraction

Total RNA was extracted from 100 μL of VTM suspension of each swab with the RNeasy Mini Kit (Qiagen, Germany) according to the manufacturer's protocol. The RNA was eluted in 60 μL AVE buffer, of which 8 μL RNA was used as the template for RT-PCR immediately, or stored at −80°C until use.

### Pan-coronavirus RT-PCR

Total RNA extracted from the rectal swab suspension was screened for the presence of coronavirus RNA using pan-coronavirus RT-PCR with universal degenerate primers. The primers were designed from a highly conserved region of the RdRp (primer sequences are presented in S4 Table). After the reverse transcription and synthesization of cDNA with SuperScript III Reverse Transcriptase (Invitrogen, United States), a semi-nested PCR was performed. The expected amplicons of two rounds were 299 bp (using primers panCoVs-OF and panCoVs-OR) and 228 bp (using primers panCoVs-IF and panCoVs-OR) in length, respectively. All positive results were repeated and confirmed with fresh RNA extracts from the original bat rectal swab suspensions. Purified DNA amplicons (both rounds) were sequenced bi-directionally with pan-coronavirus sequencing primers (S4 Table) on an ABI Prism 3730 automated capillary sequencer (Applied Biosystems, United States).

### Complete genome sequencing

Fresh RNA was extracted from sample numbers 346 and 356 which were confirmed as coronavirus positive. The RNA were subjected to Next Generation Sequencing (NGS) using the Ion Proton platform. The original NGS data were filtered, refined and mapped to the reference sequence of Ro-BatCoV HKU9 (GenBank accession number NC_009021) using SOAP (Short Oligonucleotide Alignment Program) [52]. Any remaining gaps in the genome were closed by PCR amplification of these regions with specific primers and then sequenced. Complete genome sequences were confirmed with Sanger sequencing on the fragments amplified with a set of primers that covered the whole genome. The 5’- and 3’-RACE analyses were performed with 5’- and 3’- Full RACE Kit (Takara, Japan) according to the manufacturer’s instructions.

### Genome analyses

As the amplification of 5’-end of the genome of Ro-BatCoV GCCDC1 strain 346 was unsuccessful, we focused our genome analyses on the complete genome of Ro-BatCoV GCCDC1 strain 356. This genome was compared to those of eight complete genomes of Ro-BatCoV HKU9 (GenBank accession numbers NC_009021, EF065514, EF065515, EF065516, HM211098, HM211099, HM211100 and HM211101, respectively) to annotate the 1ab, S, NS3, E, M and N ORFs, respectively. As the origin of the ORFs at the 3’-end of the genome were uncertain they were also blasted (tblastx) against the GenBank database. The amino acid sequence of ORF1ab was aligned with the reference sequences of SARS-CoV, human coronavirus HKU1, infectious bronchitis virus, turkey coronavirus, bovine coronavirus, mouse hepatitis virus and porcine epidemic diarrhea virus (GenBank accession numbers NC_004718, NC_006577, NC_001451, NC_010800, NC_003045, NC_001846 and NC_003436, respectively) to determine the cleavage and recognition patterns of the C-like proteinase and papain-like proteinase of the 16 nonstructural proteins. In addition, the sequences of the 5’ untranslated region (5’-UTR) and 3’ untranslated region (3’-UTR) were defined, and the leader sequence, the leader and body TRSs were illustrated, based on comparison with SARS-CoV.

### Confirmation of the p10 gene

To eliminate the possibility of false amplification of DNA polymerase or inaccurate assembly of NGS data, the raw NGS data were further scrutinized and reads extracted for mapping to check the continuity of the p10 sequence, especially the upstream junction site between N and p10 genes and the downstream junction site between the p10 and NS7a genes. In addition, two sets of specific primers were designed to confirm the integrity and continuity of sequence surrounding the p10 gene (primer sequences shown in S5 Table). The amplicons were subsequently cloned into the pMD18-T vector and recombinant plasmids were subjected to Sanger sequencing.

### Phylogenetic analyses

To determine the phylogenetic position of the newly identified coronavirus among the known diversity of coronaviruses, the amino acid sequences of the RdRp, S, and N proteins were used for phylogenetic analyses (GenBank accession numbers shown in S2 Table). In the case of the imported p10 gene, homologous sequences of orthoreoviruses were utilized as the background data set in the phylogenetic analysis (GenBank accession numbers listed in S3 Table). All amino acid sequences were aligned using MUSCLE [53], and all poorly or ambiguously aligned regions were removed using GBlocks [54]. Because of the short length of the p10 and N amino acid sequence alignments, more relaxed GBlocks parameters were used in these cases. In all cases phylogenetic trees of amino acid sequence alignments were inferred using the maximum likelihood method available in the PhyML package [55], with bootstrap values estimated from 1,000 replicate trees. Each tree was inferred using the LG model of amino acid substitution with values of the gamma shape parameter inferred using ProtTest [56]. Finally, all phylogenetic trees were displayed and annotated with FigTree.

### Virus culture and attempted virus isolations

Samples positive for coronavirus were cultured in Vero E6, BHK-21, MDCK, A549, HEp-2, CaCo-2 cells, as well as in an immortalized kidney cell line of *Myotis Davidii*. The cell lines were inoculated with positive samples and three blind passages were performed for each sample. The culture supernatant and cell pellet of each passage were harvested. The detection of viral replication was conducted using specific primers targeting the conserved region of RdRp.

### Subgenome identification and sequencing

Nested subgenomic mRNAs are generated during the replication cycle of coronaviruses. Hence, the identification of subgenomic mRNAs in the samples provides strong evidence for the replication of coronavirus. To analyze the possibility of replication in the newly identified bat coronavirus, primers were designed to determine the presence of viral subgenomic mRNAs in the coronavirus-positive bat rectal swab samples. Forward primers were designed targeting the leader sequence at the 5’-end of the complete genome and the putative subgenomic mRNAs, while reverse primers were designed within the ORFs or downstream of the corresponding gene (primer sequences are shown in S6 Table). Specific amplicons, that matched the expected length, were purified and then cloned into the pMD-18T vector for sequencing, while the additional suspected bands on the agarose gels were excised, purified, and then subjected to direct sequencing. Since the specific amplicon of subgenomic mRNA NS7c failed to be cloned into the vectors, the PCR product was used as a template for a second round of nested PCR. The product was then confirmed with agarose gel electrophoresis and the band was cloned for sequencing.

### p10 antiserum production

The protein family of the putative p10 protein was analyzed using PFAM [57] and InterProScan [58]. Prediction of transmembrane domains was performed using TMHMM [59], TMpred and PredictProtein [60]. Peptides corresponding to the ectodomain (from amino acid positions 2–37) and the cytoplasmic domain (the last 33 amino acids) (peptide sequences are described in S7 Table) of the putative p10 protein were synthesized (Xuheyuan Biological Technology Co., LTD, Beijing, China). After conjugation with keyhole limpet hemocyanin (KLM), the synthesized peptides were used to immunize mice for antibody production. The mice (five mice per peptide) were injected intramuscularly at their hind legs with 20 μg of the conjugated peptide mixed with adjuvant, followed by boosts until 14 days with the same conjugated peptides. Seven days after the boosts, the mice were killed and their blood collected to isolate sera. Antibody titers were determined using enzyme-linked immunosorbent assay (ELISA).

### Syncytial analysis and cell staining

In the cells infected with avian- or bat-origin fusogenic orthoreoviruses, the formation of cell syncytia depends on a p10 protein, which is encoded by the first ORF in segment S1 of the reovirus genome. It was previously demonstrated that amino acid residues of p10 proteins could be sorted into absolutely, highly, moderately and non-conserved amino acids [26].

Sequence and phylogenetic analyses indicated that the p10 gene of Ro-BatCoV GCCDC1 most likely originated in an orthoreovirus. Comparative sequence analysis revealed that although the majority of key amino acids and motifs of the Ro-BatCoV GCCDC1 p10 protein were conserved, there were 8 amino acids differences (including 2 deletions) among the 28 so-called ‘absolutely conserved’ amino acids that characterize members of the FAST family (Fig 7). Hence, it is necessary to explore the potential role of the p10 gene during the life cycle of Ro-BatCoV GCCDC1.

To determine whether the putative p10 protein could play the same role as homologous proteins of avian and bat orthoreoviruses, the p10 gene was cloned into the pCAGGS vector and the recombinant plasmid (Fig 8A) was then transfected into BHK-21 cells using Polyethylimine (PEI, Polysciences Inc.) according to the manufacturer’s protocol. At the appropriate time post-transfection, cell-to-cell fusion was observed for the syncytium formation under the light microscope using Wright-Giemsa staining and an indirect immunofluorescence assay employing the polyclonal antibodies prepared above. The p10 gene of Pulau virus, a bat orthoreovirus [49], was also cloned into the pCAGGS vector to serve as a positive control. Cells transfected with an empty pCAGGS vector were used as a mock control.

The next step is to confirm that p10 can be transcribed or translated during the replication cycle of Ro-BatCoV GCCDC1. We confirmed that the p10 gene could be transcribed from the genome during the replication cycle of Ro-BatCoV GCCDC1, with the p10 subgenomic mRNA representing a distinct signal. Further, we cloned the deduced p10 subgenome into a pcDNA3.0-derived vector to construct an artificial plasmid (Fig 8C), which could be transcribed out of an mRNA that is consistent with the p10 subgenomic mRNA in the infected cells of the host. The recombinant plasmid was transfected into BHK-21 cells and cell syncytia were observed as described above. The recombinant plasmid of Pulau virus p10 gene was still served as positive control. Cells transfected with empty pcDNA3.0 vector were used as mock control.

### Mutational analysis of the p10 protein of Ro-BatCoV GCCDC1

As the p10 protein of Ro-BatCoV GCCDC1 is the first reported in an enveloped virus, we first tried to define the key amino acids for the p10 protein in the Ro-BatCoV GCCDC1 as described previously for p10 protein of reoviruses [24, 25]. For syncytial indexing of six mutant constructs, each well of BHK-21 monolayer cells in a 6-well plate were transfected with 2 μg of plasmid DNA using Polyethylimine (PEI, Polysciences Inc.) and incubated for 5 h before replacing the transfection mixture with DMEM growth media (Invitrogen) supplemented with 10% fetal bovine serum (GIBCO). Transfected cells were paraformaldehyde-fixed and stained with Wright-Giemsa at the indicated times, and syncytia were observed and pictures were taken at ×100 magnification on an Olympus IX51FL+DP70 microscope.

### Western blotting (WB)

The original specimens of bat rectal swabs and feces were used to test the expression of p10. BHK-21 cells with transient expression plasmid of p10 gene (pCAGGS-p10) were used as a positive control. Briefly, bat specimens and BHK-21 cell lysates were subjected to SDS-PAGE and transferred to a PVDF membrane. The membranes were blocked with a 5% non-fat dry milk solution and incubated with p10 antibody overnight at 4°C followed by peroxidase-conjugated affinipure goat anti-mouse IgG (H+L) (Zhongshan Goldenbridge, Beijing). After washing with TBS-T buffer, the membrane was treated with ImmobilonTM Western Chemiluminescent HRP Substrate (Millipore, Billerica) and pictures were taken with Chemiluminescence System MicroChemi 4.2 (DNR Bio-Imaging Systems Ltd, USA).

### Accession numbers

The complete genome sequences of Ro-BatCoV GCCDC1 strains 346 and 356 have been deposited in the GenBank database and assigned accession numbers KU762337 and KU762338, respectively. We also deposited the sequences of the p10 genes from the rectal swabs of 24 bats in GenBank. All these accession numbers are listed in S8 Table.

## Supporting Information

S1 Fig Identification of p10 gene with raw NGS data. The READs were extracted from the raw NGS data and then mapped to the complete genome of Ro-BatCoV GCCDC1 using Geneious R9 (Biomatters Limited) to confirm the integrity and continuity of context sequence surrounding the p10 gene, especially the upstream junction site between N and p10 genes, and downstream junction site between p10 and NS7a genes.
(TIF)

S2 Fig Comparison of 3'-end of the coronavirus genomes from group D in the genus *Betacoronavirus*. The TRSs of NS7a or ORFx gene just downstream of the N gene are marked with red arrows. The length of intergenic spacer between N gene and NS7a or ORFx gene is indicated with numbers. GenBank accession numbers of the coronaviruses used in this analysis: Ro-BatCoV HKU9: Rousettus bat coronavirus HKU9 (NC_009021, EF065514, EF065515, EF065516, HM211098, HM211099, HM211100, HM211101); BatCoV philippines: Bat coronavirus Philippines/Diliman1525G2/2008 (AB543561); Ei-BatCoV Kenya: Eidolon bat coronavirus/Kenya/KY24/2006 (HQ728482); Ro-BatCoV Kenya: Rousettus bat coronavirus/Kenya/KY06/2006 (HQ728483).
(TIF)

S3 Fig Sequencing of subgenomic mRNAs and the identification of TRS. Amplicon of each subgenomic mRNA, including S, NS3, E, M, N, p10, NS7a, NS7b and NS7c, was sequenced and illustrated with sequencing peak pattern. The leader sequence, TRS and CDS are marked with blue, red and yellow arrow respectively. The bias of TRS of p10 gene is marked with a yellow block.
(TIF)

S4 Fig Cell syncytia with images stained DAPI and with merged images. (A) Transient expression of p10 gene and syncytium formation. **First row:** cells were stained with DAPI. **Second row:** the merged image. (From the second to the fourth row, stained monolayers were imaged using a Nikon DIAPHOT-TMD under 200× magnification. Scale bars = 50 μm). (B) Transient expression of p10 gene and syncytium formation with recombinant subgenomic p10 plasmid. **First row:** cells were stained with DAPI. **Second row:** the merged image. (From the second to forth row, stained monolayers were imaged using a Nikon DIAPHOT-TMD under 200× magnification. Scale bars = 50 μm).
(TIF)

S5 Fig Functionality of p10 in Ro-BatCoV GCCDC1 depends on traditional conserved domains relevant for membrane fusion. (A) Schematic representation of p10 protein and mutant constructs. The substituted and inserted amino acids were marked with yellow and green blocks respectively. Motifs presented in the ectodomain (HP, hydrophobic patch; CM, conserved motif), endodomain (PB, polybasic) and the central transmembrane domain (TMD) are depicted with yellow rectangles. The four conserved cysteine residues (C) are shown. The two cysteines in the ectodomain form an intra-molecular disulfide bond. (B) Transient expression and the observation of syncytium formation on the monolayer BHK-21 cells transfected with recombinant plasmid of wild type of Ro-BatCoV GCCDC1 p10 gene, mutant constructs and empty pCAGGS vector. (Wright-Giemsa staining: stained monolayers were imaged using an Olympus IX51FL+DP70 microscope under 100× magnification, scale bars = 200 μm).
(TIF)

S6 Fig Physiologically expression of p10 by Ro-BatCoV GCCDC1 virus. The expression of p10 protein are observed in fecal samples revealed by Western blotting. Rectal Swab 118: Representative of the samples which are negative for the Ro-BatCoV GCCDC1 RNA test. BHK21-p10: BHK-21 cells transfected with transient expression plasmid of p10 gene (pCAGGS-p10) as positive control. Feces 341 and 348: Feces samples of representatives of the feces samples whose corresponding rectal swabs are positive for the Ro-BatCoV GCCDC1 RNA test. Con. Swabs: The concentrated sample of 47 swabs which are positive for the Ro-BatCoV GCCDC1 RNA test.
(TIF)

S1 Table Comparison of accessory genes at the 3’-end of Ro-BatCoV GCCDC1 genome of with those of Ro-BatCoV HKU9 strains and other related bat coronaviruses. (DOCX)

S2 Table The selected coronaviruses and related GenBank accession numbers used in the construction of phylogenetic trees of RdRp, Spike and Nucleocapsid proteins. (DOCX)

S3 Table The selected orthoreoviruses and related GenBank accession numbers used in the construction of phylogenetic tree of p10 protein. (DOCX)

S4 Table Universal degenerate primers for pan-coronavirus RT-PCR and primers for sequencing. (DOCX)

S5 Table Primers for the confirmation of integrity and continuity of context sequence surrounding the p10 gene. (DOCX)

S6 Table Primers for the detection of viral subgenomic mRNAs. (DOCX)

S7 Table Peptides of putative p10 protein for antibody production. (DOCX)

S8 Table The sequences and accession numbers in this study. (DOCX)

S1 File The reads of p10 in raw data of NGS. (RAR)

S2 File Confirmation of p10 gene by cloning. (RAR)
