# Supplementary figures and images for: A Bat-Derived Putative Cross-Family Recombinant Coronavirus with a Reovirus Gene
Source: PLoS Pathog. 2016 Sep 27;12(9):e1005883. doi: 10.1371/journal.ppat.1005883 (PMC5038965; doi:10.1371/journal.ppat.1005883)

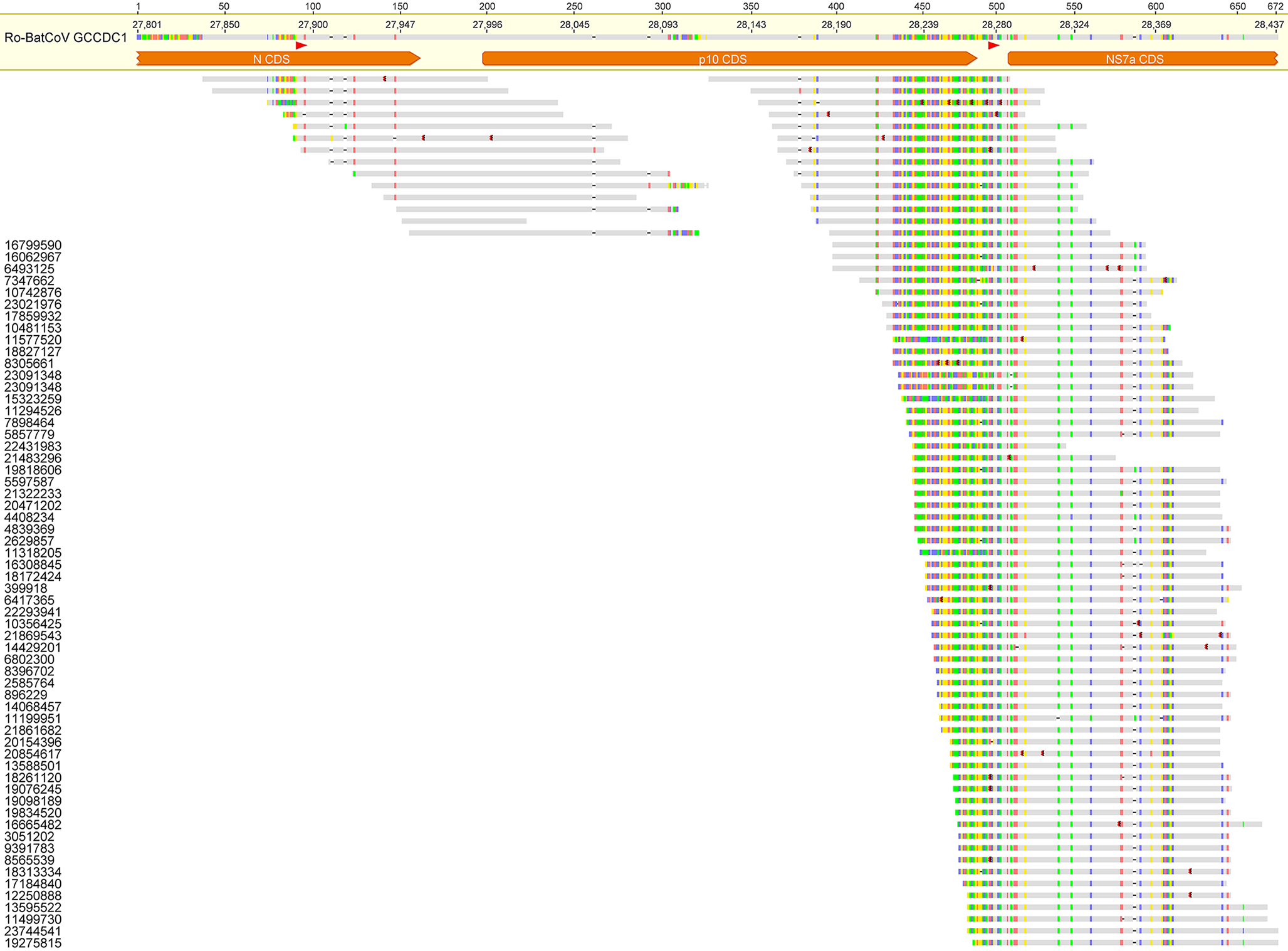

Supplement: S1 Fig — The READs were extracted from the raw NGS data and then mapped to the complete genome of Ro-BatCoV GCCDC1 using Geneious R9 (Biomatters Limited) to confirm the integrity and continuity of context sequence surrounding the p10 gene, especially the upstream junction site between N and p10 genes, and downstream junction site between p10 and NS7a genes. (TIF) [file ppat.1005883.s001.tif]

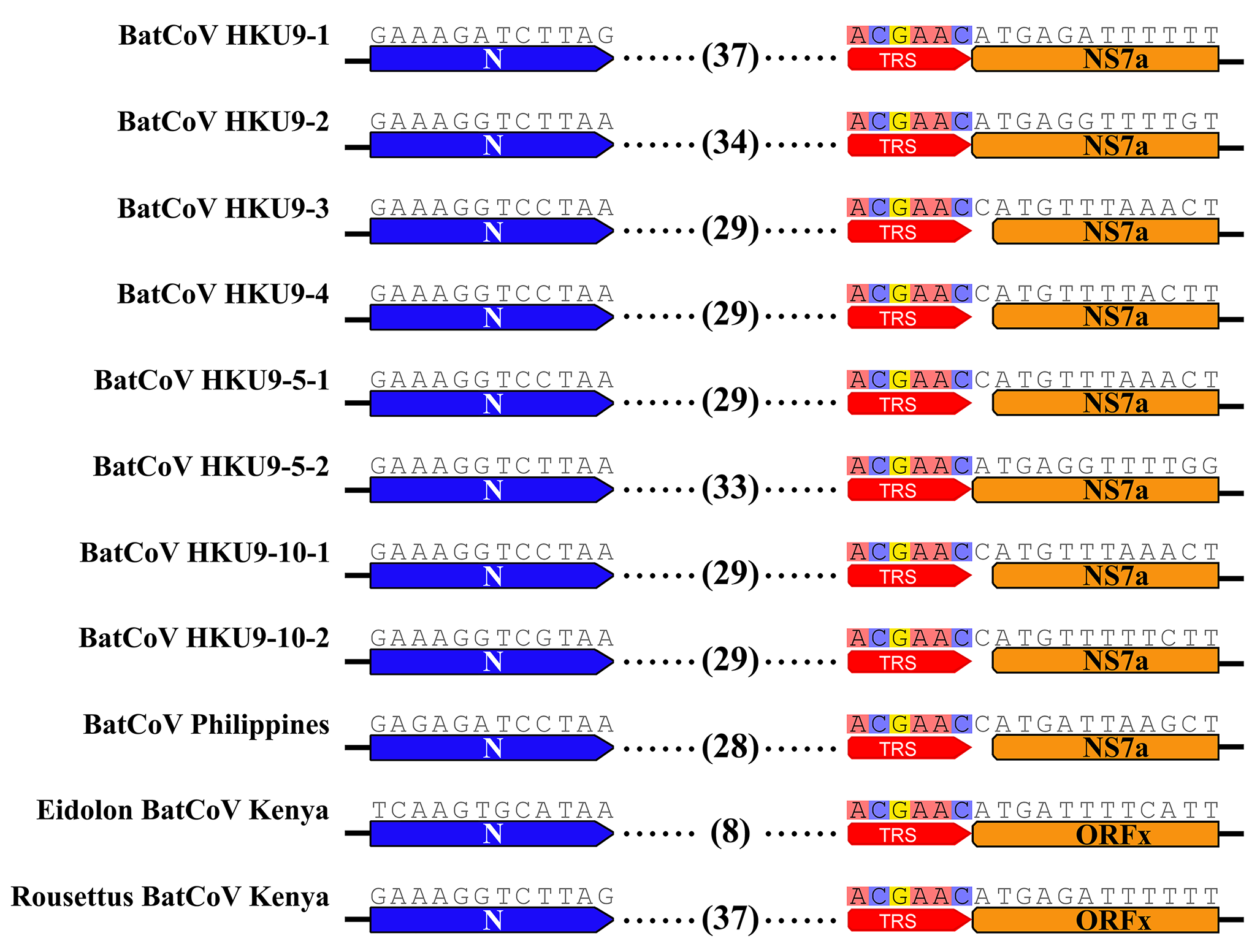

Supplement: S2 Fig — The TRSs of NS7a or ORFx gene just downstream of the N gene are marked with red arrows. The length of intergenic spacer between N gene and NS7a or ORFx gene is indicated with numbers. GenBank accession numbers of the coronaviruses used in this analysis: Ro-BatCoV HKU9: Rousettus bat coronavirus HKU9 (NC_009021, EF065514, EF065515, EF065516, HM211098, HM211099, HM211100, HM211101); BatCoV philippines: Bat coronavirus Philippines/Diliman1525G2/2008 (AB543561); Ei-BatCoV Kenya: Eidolon bat coronavirus/Kenya/KY24/2006 (HQ728482); Ro-BatCoV Kenya: Rousettus bat coronavirus/Kenya/KY06/2006 (HQ728483). (TIF) [file ppat.1005883.s002.tif]

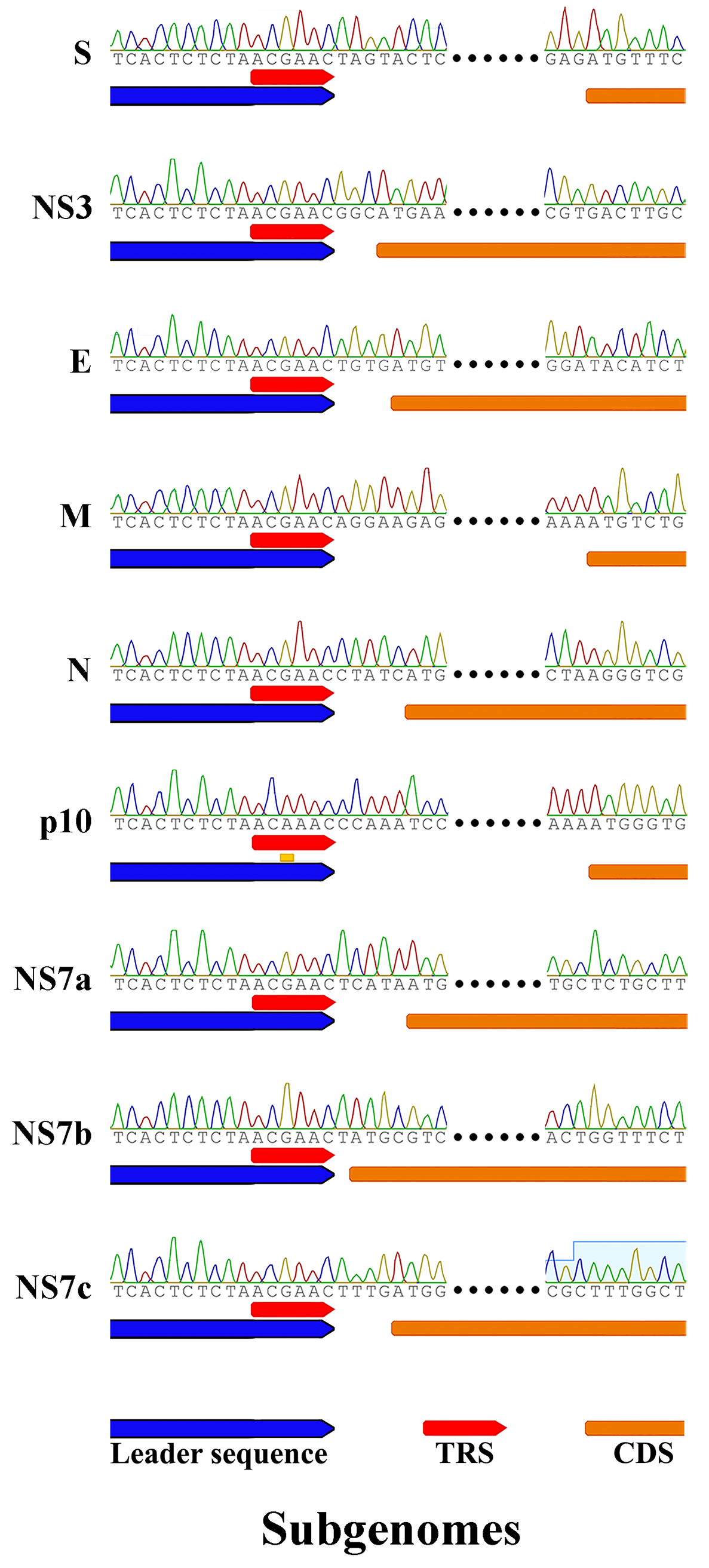

Supplement: S3 Fig — Amplicon of each subgenomic mRNA, including S, NS3, E, M, N, p10, NS7a, NS7b and NS7c, was sequenced and illustrated with sequencing peak pattern. The leader sequence, TRS and CDS are marked with blue, red and yellow arrow respectively. The bias of TRS of p10 gene is marked with a yellow block. (TIF) [file ppat.1005883.s003.tif]

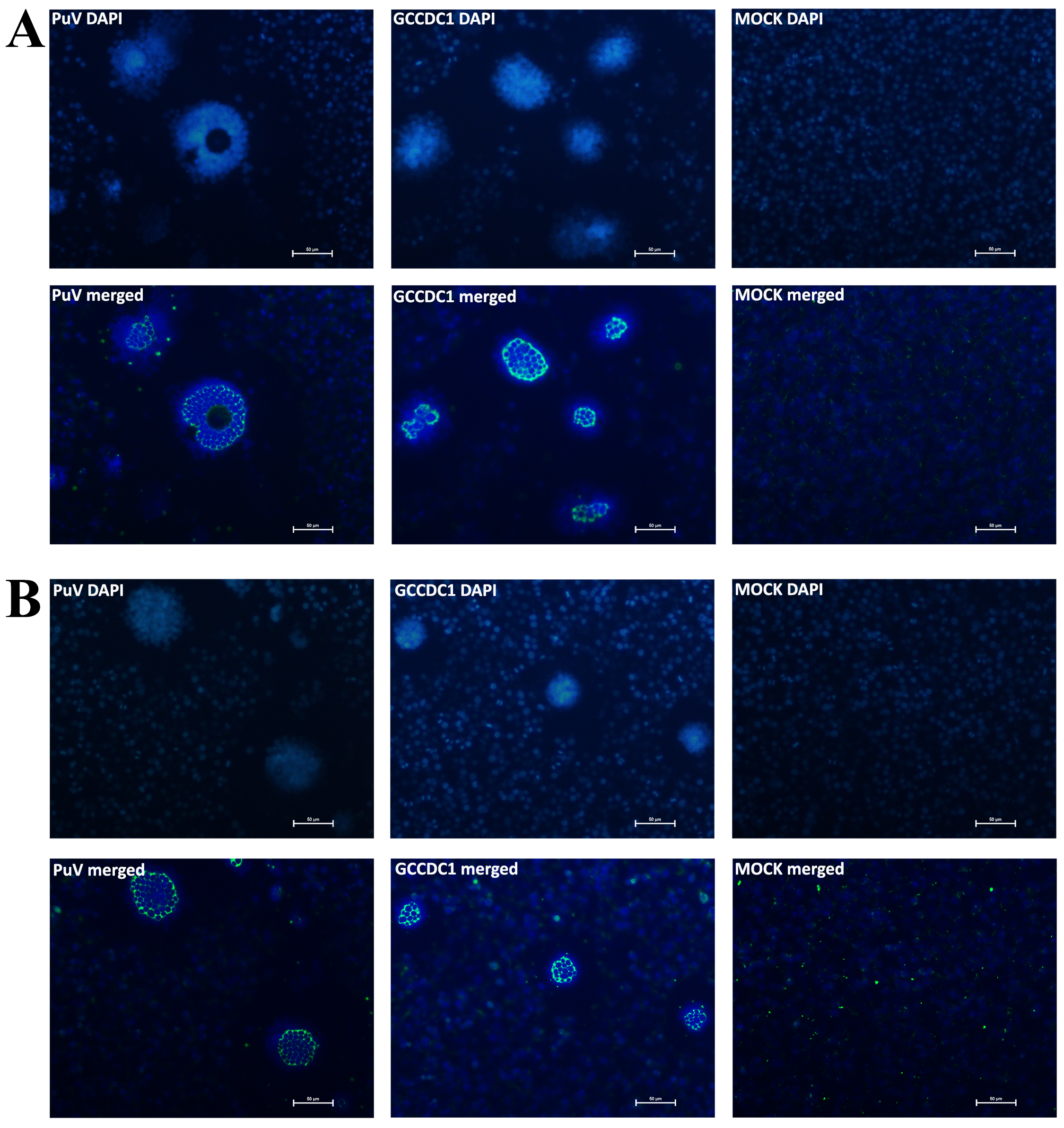

Supplement: S4 Fig — (A) Transient expression of p10 gene and syncytium formation. First row: cells were stained with DAPI. Second row: the merged image. (From the second to the fourth row, stained monolayers were imaged using a Nikon DIAPHOT-TMD under 200× magnification. Scale bars = 50 μm). (B) Transient expression of p10 gene and syncytium formation with recombinant subgenomic p10 plasmid. First row: cells were stained with DAPI. Second row: the merged image. (From the second to forth row, stained monolayers were imaged using a Nikon DIAPHOT-TMD under 200× magnification. Scale bars = 50 μm). (TIF) [file ppat.1005883.s004.tif]

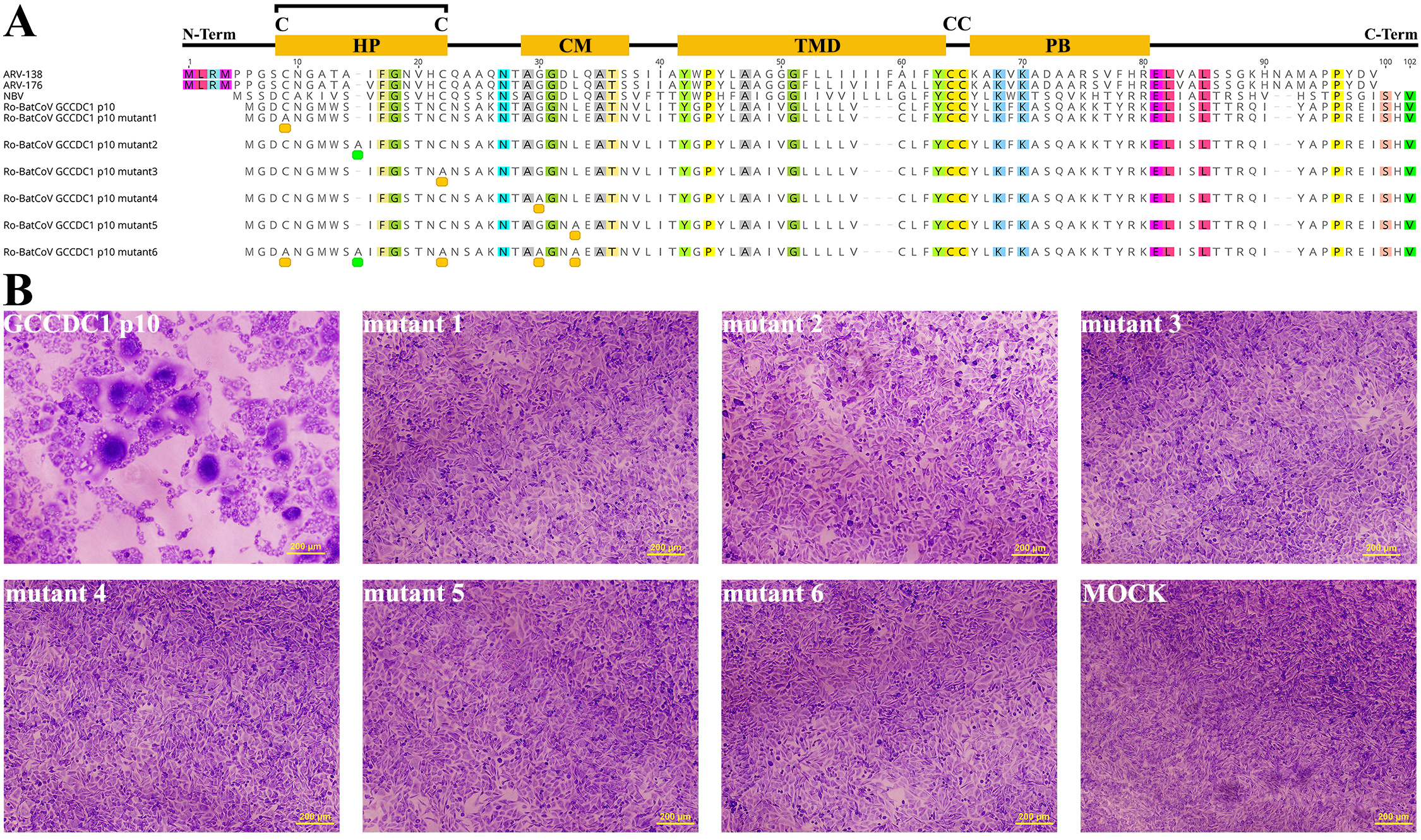

Supplement: S5 Fig — (A) Schematic representation of p10 protein and mutant constructs. The substituted and inserted amino acids were marked with yellow and green blocks respectively. Motifs presented in the ectodomain (HP, hydrophobic patch; CM, conserved motif), endodomain (PB, polybasic) and the central transmembrane domain (TMD) are depicted with yellow rectangles. The four conserved cysteine residues (C) are shown. The two cysteines in the ectodomain form an intra-molecular disulfide bond. (B) Transient expression and the observation of syncytium formation on the monolayer BHK-21 cells transfected with recombinant plasmid of wild type of Ro-BatCoV GCCDC1 p10 gene, mutant constructs and empty pCAGGS vector. (Wright-Giemsa staining: stained monolayers were imaged using an Olympus IX51FL+DP70 microscope under 100× magnification, scale bars = 200 μm). (TIF) [file ppat.1005883.s005.tif]

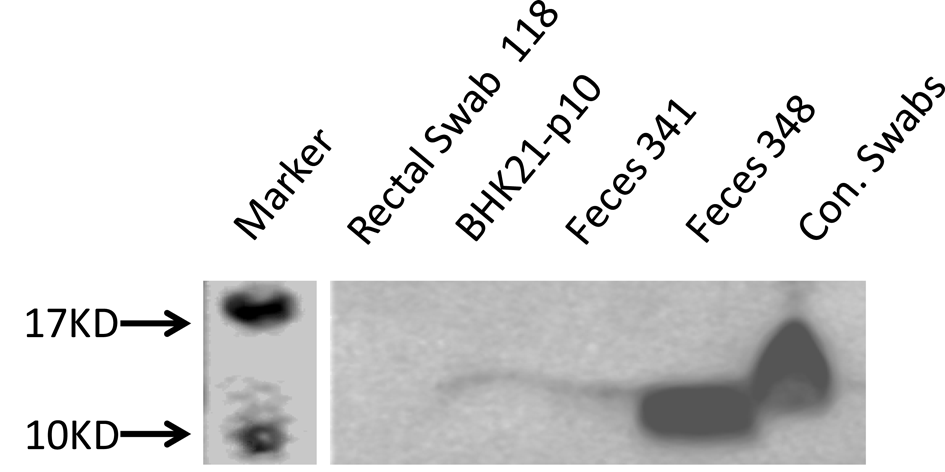

Supplement: S6 Fig — The expression of p10 protein are observed in fecal samples revealed by Western blotting. Rectal Swab 118: Representative of the samples which are negative for the Ro-BatCoV GCCDC1 RNA test. BHK21-p10: BHK-21 cells transfected with transient expression plasmid of p10 gene (pCAGGS-p10) as positive control. Feces 341 and 348: Feces samples of representatives of the feces samples whose corresponding rectal swabs are positive for the Ro-BatCoV GCCDC1 RNA test. Con. Swabs: The concentrated sample of 47 swabs which are positive for the Ro-BatCoV GCCDC1 RNA test. (TIF) [file ppat.1005883.s006.tif]
