# Supplementary material for: A Bat-Derived Putative Cross-Family Recombinant Coronavirus with a Reovirus Gene
Source: PLoS Pathog. 2016 Sep 27;12(9):e1005883. doi: 10.1371/journal.ppat.1005883 (PMC5038965; doi:10.1371/journal.ppat.1005883)
Supplement: S1 Table — (DOCX) [file ppat.1005883.s007.docx]

**S1 Table. Comparison of accessory genes at the 3’-end of Ro-BatCoV GCCDC1 genome of with those of Ro-BatCoV HKU9 strains and other related bat coronaviruses.**

|  |  | **Ro-BatCoV GCCDC1 strain 356 (identities/cover)** | | |
| --- | --- | --- | --- | --- |
|  |  | **NS7a** | **NS7b** | **NS7c** |
| Ro-BatCoV HKU9-1 | NS7a | 40% (87%) |  |  |
|  | NS7b |  |  | 53% (99%) |
| Ro-BatCoV HKU9-2 | NS7a |  |  |  |
|  | NS7b |  |  | 30% (90%) |
| Ro-BatCoV HKU9-3 | NS7a |  | 33% (90%) |  |
|  | NS7b |  |  | 32% (99%) |
| Ro-BatCoV HKU9-4 | NS7a |  | 32% (91%) |  |
|  | NS7b |  |  | 32% (95%) |
| Ro-BatCoV HKU9-5-1 | NS7a |  |  |  |
|  | NS7b |  |  |  |
| Ro-BatCoV HKU9-5-2 | NS7a |  |  |  |
|  | NS7b |  |  |  |
| Ro-BatCoV HKU9-10-1 | NS7a |  | 33% (90%) |  |
|  | NS7b |  |  | 32% (99%) |
| Ro-BatCoV HKU9-10-2 | NS7a |  | 42% (91%) |  |
|  | NS7b |  |  | 36% (99%) |
| Ei-BatCoV Kenya | ORF7x |  |  |  |
|  | ORF7y |  |  | 29% (85%) |
| Ro-BatCoV Kenya | ORF7x | 40% (87%) |  |  |
|  | ORF7y |  |  | 52% (99%) |
| BatCoV Philippines | NS7a |  | 35% (88%) |  |
|  | NS7b |  |  |  |
|  | NS7c |  |  | 29% (85%) |

There are two ORFs just downstream of N gene of Ro-BatCoV HKU9 strains and BatCoV Philippines, which were named NS7a and NS7b respectively, Ei-BatCoV Kenya and Ro-BatCoV Kenya, which were named NS7x and NS7y respectively. However, clearly there are four ORFs just downstream of N gene of Ro-BatCoV GCCDC1. According to the conventional rule, the second to forth ORF was designated NS7a, NS7b and NS7c respectively. The amino acid identities of these genes were determined with blastp on the NCBI website. GenBank accession numbers of the viruses used in this analysis: Ro-BatCoV HKU9: Rousettus bat coronavirus HKU9 (NC_009021, EF065514, EF065515, EF065516, HM211098, HM211099, HM211100, HM211101); BatCoV philippines: Bat coronavirus Philippines/Diliman1525G2/2008 (AB543561); Ei-BatCoV Kenya: Eidolon bat coronavirus/Kenya/KY24/2006 (HQ728482); Ro-BatCoV Kenya: Rousettus bat coronavirus/Kenya/KY06/2006 (HQ728483).
