# Supplementary material for: A Bat-Derived Putative Cross-Family Recombinant Coronavirus with a Reovirus Gene
Source: PLoS Pathog. 2016 Sep 27;12(9):e1005883. doi: 10.1371/journal.ppat.1005883 (PMC5038965; doi:10.1371/journal.ppat.1005883)
Supplement: S2 Table — (DOCX) [file ppat.1005883.s008.docx]

**S2 Table. The selected coronaviruses and related GenBank accession numbers used in the construction of phylogenetic trees of RdRp, Spike and Nucleocapsid proteins.**

| Beluga Whale coronavirus SW1: NC_010646 | Avian infectious bronchitis virus: NC_001451 |
| --- | --- |
| Turkey coronavirus: NC_010800 | Feline infectious peritonitis virus: NC_002306 |
| Bat coronavirus 1A: NC_010437 | Bat coronavirus HKU8: NC_010438 |
| Porcine epidemic diarrhea virus: NC_003436 | Scotophilus bat coronavirus 512: NC_009657 |
| Human coronavirus NL63: NC_005831 | Human coronavirus 229E: NC_002645 |
| Bat coronavirus HKU2: NC_009988 | Murine hepatitis virus strain A59: NC_001846 |
| Human coronavirus HKU1: NC_006577 | Dromedary camel coronavirus HKU23 strain HKU23-265F: KF906249 |
| Human coronavirus OC43: NC_005147 | Bat coronavirus HKU4-1: NC_009019 |
| Middle East respiratory syndrome coronavirus: NC_019843 | Bat coronavirus HKU5-1: NC_009020 |
| Bat SARS coronavirus HKU3-1: DQ022305 | SARS coronavirus: NC_004718 |
| Eidolon bat coronavirus/Kenya/KY24/2006: HQ728482 | Bat coronavirus HKU9-10-2: HM211101 |
| Bat coronavirus HKU9-5-2: HM211099 | Bat coronavirus HKU9-2: EF065514 |
| Bat coronavirus HKU9-3: EF065515 | Bat coronavirus HKU9-5-1: HM211098 |
| Bat coronavirus HKU9-10-1: HM211100 | Bat coronavirus HKU9-4: EF065516 |
| Rousettus bat coronavirus/Kenya/KY06/2006: HQ728483 | Bat coronavirus HKU9-1: NC_009021 |
| Bulbul coronavirus HKU11-934: FJ376619 | Thrush coronavirus HKU12-600: NC_011549 |
| Munia coronavirus HKU13: NC_011550 | Porcine coronavirus HKU15: NC_016990 |

BWCoV SW1: Beluga Whale coronavirus SW1; IBV: Avian infectious bronchitis virus; TCoV: Turkey coronavirus; FIPV: Feline infectious peritonitis virus; Mi-BatCoV 1A: Miniopterus bat coronavirus 1A; Mi-BatCoV HKU8: Miniopterus bat coronavirus HKU8; PEDV: Porcine epidemic diarrhea virus; Sc-BatCoV 512: Scotophilus bat coronavirus 512; HCoV-NL63: Human coronavirus NL63; HCoV-229E: Human coronavirus 229E; Rh-BatCoV HKU2: Rhinolophus bat coronavirus HKU2; MHV: Murine hepatitis virus strain A59; HCoV-HKU1: Human coronavirus HKU1; DcCoV UAE-HKU23: Dromedary camel coronavirus HKU23 strain HKU23-265F; HCoV-OC43: Human coronavirus OC43; Ty-BatCoV HKU4: Tylonycteris bat coronavirus HKU4; MERS-CoV: Middle East respiratory syndrome coronavirus; Pi-BatCoV HKU5: Pipistrellus bat coronavirus HKU5; SARSr-Rh-BatCoV HKU3: SARS-related Rhinolophus bat coronavirus HKU3; SARS-CoV: SARS coronavirus; Ei-BatCoV Kenya: Eidolon bat coronavirus/Kenya/KY24/2006; Ro-BatCoV HKU9-10-2: Rousettus Bat coronavirus HKU9-10-2; Ro-BatCoV HKU9-5-2: Rousettus Bat coronavirus HKU9-5-2; Ro-BatCoV HKU9-2: Rousettus Bat coronavirus HKU9-2; Ro-BatCoV HKU9-3: Rousettus Bat coronavirus HKU9-3; Ro-BatCoV HKU9-5-1: Rousettus Bat coronavirus HKU9-5-1; Ro-BatCoV HKU9-10-1: Rousettus Bat coronavirus HKU9-10-1; Ro-BatCoV HKU9-4: Rousettus Bat coronavirus HKU9-4; Ro-BatCoV Kenya: Rousettus bat coronavirus/Kenya/KY06/2006; Ro-BatCoV HKU9-1: Rousettus Bat coronavirus HKU9-1; BuCoV HKU11: Bulbul coronavirus HKU11; ThCoV HKU12: Thrush coronavirus HKU12; MunCoV HKU13: Munia coronavirus HKU13; PorCoV HKU15: porcine coronavirus HKU15;
