# Supplementary material for: A Bat-Derived Putative Cross-Family Recombinant Coronavirus with a Reovirus Gene
Source: PLoS Pathog. 2016 Sep 27;12(9):e1005883. doi: 10.1371/journal.ppat.1005883 (PMC5038965; doi:10.1371/journal.ppat.1005883)
Supplement: S3 Table — (DOCX) [file ppat.1005883.s009.docx]

**S3 Table. The selected orthoreoviruses and related GenBank accession numbers used in the construction of phylogenetic tree of p10 protein.**

| Duck reovirus strain 091: JX478256 | Duck reovirus strain TH11: KC493571 |
| --- | --- |
| Duck reovirus strain QY: KF689545 | Avian reovirus strain 176: AF218358 |
| Avian orthoreovirus strain HB06: EU526387 | Avian orthoreovirus strain GX110116: KF741752 |
| Avian orthoreovirus strain GX110058: KF741742 | Avian reovirus strain S1133: AF330703 |
| Muscovy duck reovirus strain YJL: DQ191363 | Avian orthoreovirus isolate GuangxiR1: KC183744 |
| Avian orthoreovirus strain 916: AY436604 | Avian reovirus strain 138: AF218359 |
| Orthoreovirus: OTOSIGMA | Avian orthoreovirus strain NC/98: DQ995806 |
| Avian orthoreovirus isolate NC/SEP-R108/03: DQ996606 | Avian orthoreovirus isolate NC/SEP-R61/03: DQ996608 |
| Avian orthoreovirus isolate TARV-MN2: KF872233 | Avian orthoreovirus isolate TARV-MN4: KF872235 |
| Avian orthoreovirus isolate TARV-MN7: KF872239 | Avian orthoreovirus isolate TARV-MN6: KF872237 |
| Avian orthoreovirus isolate TARV-MN3: KF872234 | Avian orthoreovirus isolate NC/PEMS/85: DQ996607 |
| Avian orthoreovirus isolate TX/99: DQ996610 | Avian orthoreovirus strain T1781: KC865792 |
| Avian orthoreovirus: AB914766 | Psittacine orthoreovirus SRK/Germany/2007: EU252582 |
| Avian orthoreovirus: HM222974 | Tvarminne avian virus: KF692095 |
| Reovirus sp. Miyazaki: AB521793 | Pulau reovirus: AY357730 |
| Cangyuan orthoreovirus strain Cangyuan: NC_025806 | Pteropine orthoreovirus Sikamat/MYS/2010: JF811580 |
| Melaka orthoreovirus: NC_020448 | Kampar orthoreovirus: EU448334 |
| Nelson bay reovirus: AF218360 | Pteropine orthoreovirus strain Indonesia/2010: KM279386 |
| Xi river reovirus: GU188274 |  |

All the orthoreoviruses selected to analysis are fusogenic avian or bat origin orthoreoviruses which contain a p10 gene in the segment S1 of the genome.
