# Supplementary material for: A Bat-Derived Putative Cross-Family Recombinant Coronavirus with a Reovirus Gene
Source: PLoS Pathog. 2016 Sep 27;12(9):e1005883. doi: 10.1371/journal.ppat.1005883 (PMC5038965; doi:10.1371/journal.ppat.1005883)
Supplement: S4 Table — (DOCX) [file ppat.1005883.s010.docx]

**S4 Table. Universal degenerate primers for pan-coronavirus RT-PCR and primers for sequencing.**

| **Primers** | **Primer sequence** |
| --- | --- |
| panCoVs-OF | 5’- TGTTATTGGAACCACGAAGTTYTAYGGNGGNTG -3’ |
| panCoVs-IF | 5’- GTTTTGTTCTTATGGGTTGGGATTAYCCNAARTGYGA -3’ |
| panCoVs-OR | 5’- TAGTAGCATCTCCGCTGCTAGTNCCNCCNGGYTT -3’ |
| panCorona-seq-1F | 5’- TGTTATTGGAACCACGAAG -3’ |
| panCorona-seq-2F | 5’- CTTATGGGTTGGGATTA -3’ |
| panCorona-seq-R | 5’- GCATCTCCGCTGCTAGT -3’ |

The primers were designed from a highly conserved region of RNA dependent RNA polymerase (RdRp) based on the alignment and analysis of all available coronavirus genomes. This set of primers are designed for semi-nested PCR, panCoVs-OF and panCoVs-OR are used for the first round, the expected length of amplicon is 299 bp while panCoVs-IF and panCoVs-OR are used for the second round, the expected length of amplicon is 228 bp; The amplicons of first and second round could be easily differentiated as one is up the DNA marker 250 bp, another is blow the DNA marker 250 bp; panCorona-seq-1F, panCorona-seq-2F and panCorona-seq-R are sequencing primers. panCorona-seq-1F and panCorona-seq-R are used for the sequencing of amplicon of the first round while panCorona-seq-2F and panCorona-seq-R are used for the sequencing of amplicon of the second round.
