# Supplementary material for: A Bat-Derived Putative Cross-Family Recombinant Coronavirus with a Reovirus Gene
Source: PLoS Pathog. 2016 Sep 27;12(9):e1005883. doi: 10.1371/journal.ppat.1005883 (PMC5038965; doi:10.1371/journal.ppat.1005883)
Supplement: S5 Table — (DOCX) [file ppat.1005883.s011.docx]

**S5 Table. Primers for the confirmation of integrity and continuity of context sequence surrounding the p10 gene.**

| **Primers** | **Primer sequences** | **Position on the genome of Ro-BatCoV GCCDC1 strain 356** |
| --- | --- | --- |
| CoV-Indels-1OF | TAAACTAGGCACTGACGACC | 27527 - 27546 |
| CoV-Indels-1OR | GAAATGCGGGATAGACACC | 29572 - 29590 |
| CoV-Indels-1IF | ACCTGGCTTAACTATCGTGG | 27636 - 27655 |
| CoV-Indels-1IR | TATGCAGCGGACTACATTCG | 29476 - 29495 |
| CoV-Indels-2OF | GACCTGAGATTTGAGGGTT | 26356 - 26374 |
| CoV-Indels-2OR | GCAGTAGGAGACGCATAGTT | 28860 - 28879 |
| CoV-Indels-2IF | GCGAAGACGGGGATACAATCG | 26543 - 26563 |
| CoV-Indels-2IR1 | GTCTGACAGCCGCCGACAATA | 28632 - 28652 |
| CoV-Indels-2IR2 | GCAGAGCAAGAGCAGGACAGG | 28294 - 28314 |

The primers of CoV-Indels-1OF and CoV-Indels-1OR, CoV-Indels-1IF and CoV-Indels-1IR could cover partial N gene, whole p10 gene, whole NS7a, whole NS7b and partial NS7c gene; The primers of CoV-Indels-2OF and CoV-Indels-2OR, CoV-Indels-2IF and CoV-Indels-2IR1/ CoV-Indels-2IR2 could cover partial M gene, whole N gene, whole p10 gene and partial NS7a gene.
