# Supplementary material for: A Bat-Derived Putative Cross-Family Recombinant Coronavirus with a Reovirus Gene
Source: PLoS Pathog. 2016 Sep 27;12(9):e1005883. doi: 10.1371/journal.ppat.1005883 (PMC5038965; doi:10.1371/journal.ppat.1005883)
Supplement: S6 Table — (DOCX) [file ppat.1005883.s012.docx]

**S6 Table. Primers for the detection of viral subgenomic mRNAs.**

| **Primers** | **Primer sequence** |
| --- | --- |
| TRS-5UTR-OF | 5’- GCGTGCGTGCAATCAACT -3’ |
| TRS-5UTR-IF | 5’- GATTCGTCTTGTACGATTCAC -3’ |
| TRS-S-OR | 5’- CAAGGAGCCCGTCAAAGA -3’ |
| TRS-S-IR | 5’- AGCGGTACTGAGACATAGGTAG -3’ |
| TRS-NS3-OR | 5’- TCTGTCCAGCAGCAAAATAG -3’ |
| TRS-NS3-IR | 5’- ACCGCAAAGTTGTCAGCAGT -3’ |
| TRS-E-OR | 5’- TAGTAGCTCTTCCTGTTCG -3’ |
| TRS-E-IR | 5’- TGTTGGCTTACACACTGAGG -3’ |
| TRS-M-OR | 5’- AACCCTCAAATCTCAGGTCA -3’ |
| TRS-M-IR | 5’- GCCACATAATGGACGAGATA -3’ |
| TRS-N-OR | 5’- CAGCCTGCGGTGGTAAAT -3’ |
| TRS-N-IR | 5’- CATAGCGACCAGTCCCAGT -3’ |
| TRS-p10-OR | 5’- CGCGTGGTCAAAGAAATAAG -3’ |
| TRS-p10-IR | 5’- GGTAGGTCTTCTTCGCTTGG -3’ |
| TRS-NS7a-OR | 5’- AGCGTAAAGCCGCTGGTG -3’ |
| TRS-NS7a-IR | 5’- CAACCTGCTCCAGACCACAT -3’ |
| TRS-NS7b-OR | 5’- CCTAGCAACGCGACCCTC -3’ |
| TRS-NS7b-IR | 5’- TCTTACTCCCTCCAACTTCAC -3’ |
| TRS-NS7c-OR | 5’- TTCATGGGTGGCTCTTCC -3’ |
| TRS-NS7c-IR | 5’- AACTAAAAGGTAGGGTTTATGG -3’ |

The forward primers were designed targeting the leader sequence at the 5’-end of the complete genome and the putative subgenomic mRNAs, whereas the reverse primers were designed within the ORFs or just downstream of corresponding gene. Nested PCR primers were designed in this experiment. If there is no effective amplification in the first round, the PCR products will be used as the template for the second round.
