# Supplementary material for: A Bat-Derived Putative Cross-Family Recombinant Coronavirus with a Reovirus Gene
Source: PLoS Pathog. 2016 Sep 27;12(9):e1005883. doi: 10.1371/journal.ppat.1005883 (PMC5038965; doi:10.1371/journal.ppat.1005883)
Supplement: S7 Table — (DOCX) [file ppat.1005883.s013.docx]

**S7 Table. Peptides of putative p10 protein for antibody production.**

| **peptide name** | **peptide sequence** |
| --- | --- |
| GCCDC1-p10N | GDCNGMWSIFGSTNCNSAKNTAGGNLEATNVLITYG |
| GCCDC1-p10C | KFKASQAKKTYRKELISLTTRQIYAPPREISHV |

Transmembrane domains of p10 protein was predicted by using TMHMM [[1](#_ENREF_1)], TMpred and PredictProtein [[2](#_ENREF_2)]. Peptides corresponding to the ectodomain (From 2 to 37 amino acids) and the cytoplasmic domain (the last 33 amino acids) were synthesized. Each peptide was synthesized for 20 mg with percent purity of 95%.

1. Krogh A, Larsson B, von Heijne G, Sonnhammer EL. Predicting transmembrane protein topology with a hidden Markov model: application to complete genomes. J Mol Biol. 2001;305(3):567-80. doi: 10.1006/jmbi.2000.4315. PubMed PMID: 11152613.

2. Rost B, Yachdav G, Liu J. The PredictProtein server. Nucleic Acids Res. 2004;32(Web Server issue):W321-6. doi: 10.1093/nar/gkh377. PubMed PMID: 15215403; PubMed Central PMCID: PMCPMC441515.
