# Supplementary material for: A Bat-Derived Putative Cross-Family Recombinant Coronavirus with a Reovirus Gene
Source: PLoS Pathog. 2016 Sep 27;12(9):e1005883. doi: 10.1371/journal.ppat.1005883 (PMC5038965; doi:10.1371/journal.ppat.1005883)
Supplement: S8 Table — (DOCX) [file ppat.1005883.s014.docx]

**S8 Table. The sequences and accession numbers in this study**

| **Strains:** | **Sequences:** | **Accession Numbers:** |
| --- | --- | --- |
| Ro-BatCoV GCCDC1 strain 356 | Complete genome | KU762338 |
| Ro-BatCoV GCCDC1 strain 346 | 38 nucleotides truncation at 5’-end of the genome | KU762337 |
| Ro-BatCoV GCCDC1 strain 26 | partial N gene-p10 gene-NS7a gene-partial NS7b gene | KX652379 |
| Ro-BatCoV GCCDC1 strain 30 | partial N gene-p10 gene-NS7a gene- NS7b gene | KX652380 |
| Ro-BatCoV GCCDC1 strain 51 | partial N gene-p10 gene-NS7a gene-partial NS7b gene | KX652381 |
| Ro-BatCoV GCCDC1 strain 54 | partial N gene-p10 gene-NS7a gene- NS7b gene | KX652382 |
| Ro-BatCoV GCCDC1 strain 55 | partial N gene-p10 gene-NS7a gene- NS7b gene | KX652383 |
| Ro-BatCoV GCCDC1 strain 60 | partial N gene-p10 gene-NS7a gene-partial NS7b gene | KX652384 |
| Ro-BatCoV GCCDC1 strain 84 | partial N gene-p10 gene-NS7a gene- NS7b gene | KX652385 |
| Ro-BatCoV GCCDC1 strain 86 | partial N gene-p10 gene-NS7a gene-partial NS7b gene | KX652386 |
| Ro-BatCoV GCCDC1 strain 320 | partial N gene-p10 gene-NS7a gene- NS7b gene | KX652387 |
| Ro-BatCoV GCCDC1 strain 322 | partial N gene-p10 gene-NS7a gene- NS7b gene | KX652388 |
| Ro-BatCoV GCCDC1 strain 325 | partial N gene-p10 gene-NS7a gene-partial NS7b gene | KX652389 |
| Ro-BatCoV GCCDC1 strain 330 | partial N gene-p10 gene-NS7a gene- NS7b gene | KX652390 |
| Ro-BatCoV GCCDC1 strain 336 | partial N gene-p10 gene-NS7a gene- NS7b gene | KX652391 |
| Ro-BatCoV GCCDC1 strain 337 | partial N gene-p10 gene-NS7a gene-partial NS7b gene | KX652392 |
| Ro-BatCoV GCCDC1 strain 339 | partial N gene-p10 gene-NS7a gene-partial NS7b gene | KX652393 |
| Ro-BatCoV GCCDC1 strain 347 | partial N gene-p10 gene-NS7a gene- NS7b gene | KX652394 |
| Ro-BatCoV GCCDC1 strain 348 | partial N gene-p10 gene-NS7a gene-partial NS7b gene | KX652395 |
| Ro-BatCoV GCCDC1 strain 375 | partial N gene-p10 gene-NS7a gene- NS7b gene | KX652396 |
| Ro-BatCoV GCCDC1 strain 380 | partial N gene-p10 gene-NS7a gene- NS7b gene | KX652397 |
| Ro-BatCoV GCCDC1 strain 387 | partial N gene-p10 gene-NS7a gene-partial NS7b gene | KX652398 |
| Ro-BatCoV GCCDC1 strain 390 | partial N gene-p10 gene-NS7a gene-partial NS7b gene | KX652399 |
| Ro-BatCoV GCCDC1 strain 400 | partial N gene-p10 gene-NS7a gene-partial NS7b gene | KX652400 |
| Ro-BatCoV GCCDC1 strain 407 | partial N gene-p10 gene-NS7a gene- NS7b gene | KX652401 |
| Ro-BatCoV GCCDC1 strain 454 | partial N gene-p10 gene-NS7a gene- NS7b gene | KX652402 |

**Note:** 1) Ro-BatCoV GCCDC1: Rousettus leschenaulti coronavirus GCCDC1; 2) The sequences have not been released. However, they will be available upon request.
